# Supplementary material for: A chromatin structure‐based model accurately predicts DNA replication timing in human cells
Source: Mol Syst Biol. 2014 Mar 28;10(3):722. doi: 10.1002/msb.134859 (PMC4017678; doi:10.1002/msb.134859)
Supplement: Supplementary file 1 — Supplementary Figure S1 [file MSB-10-3-722-s01.pdf]

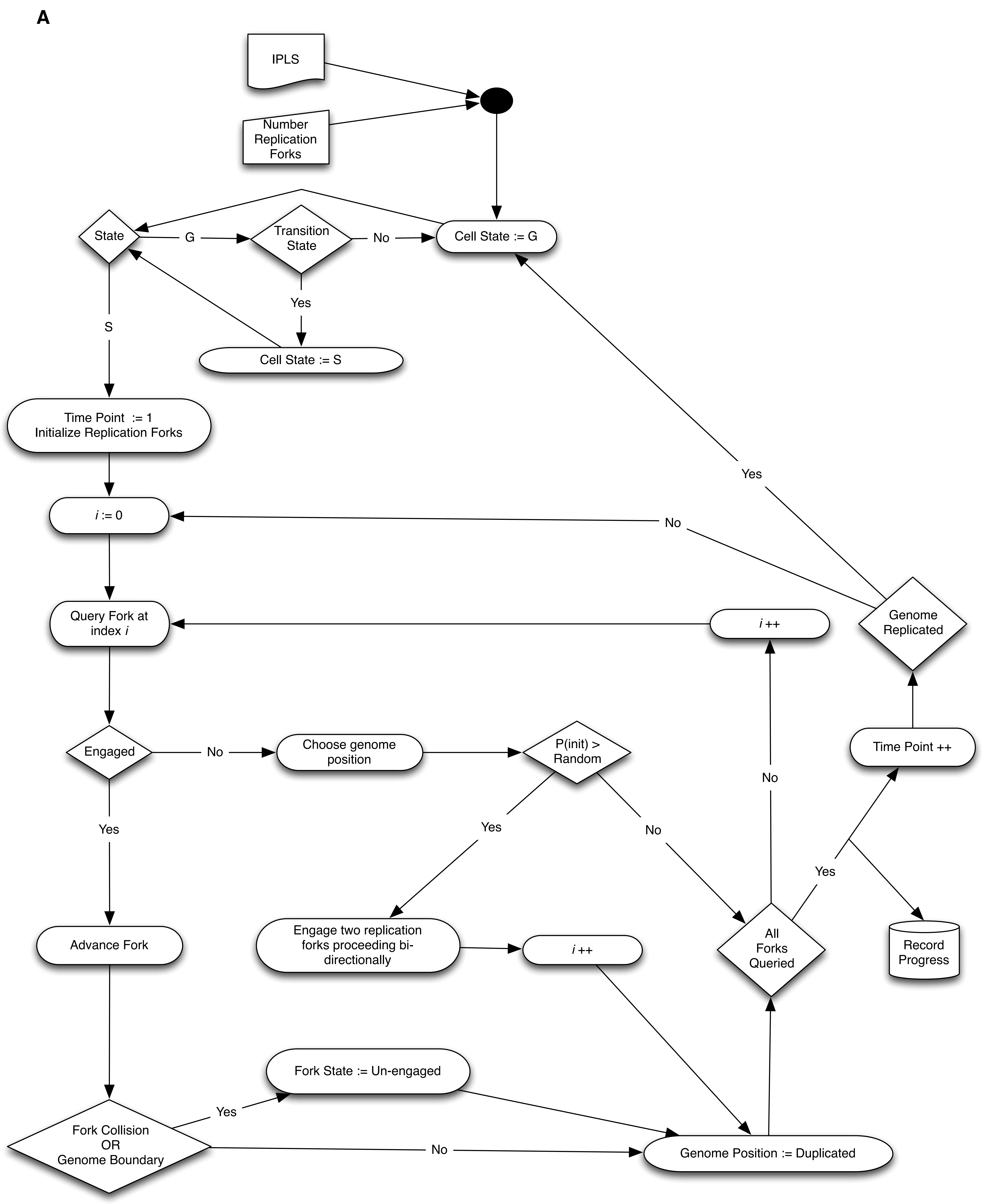

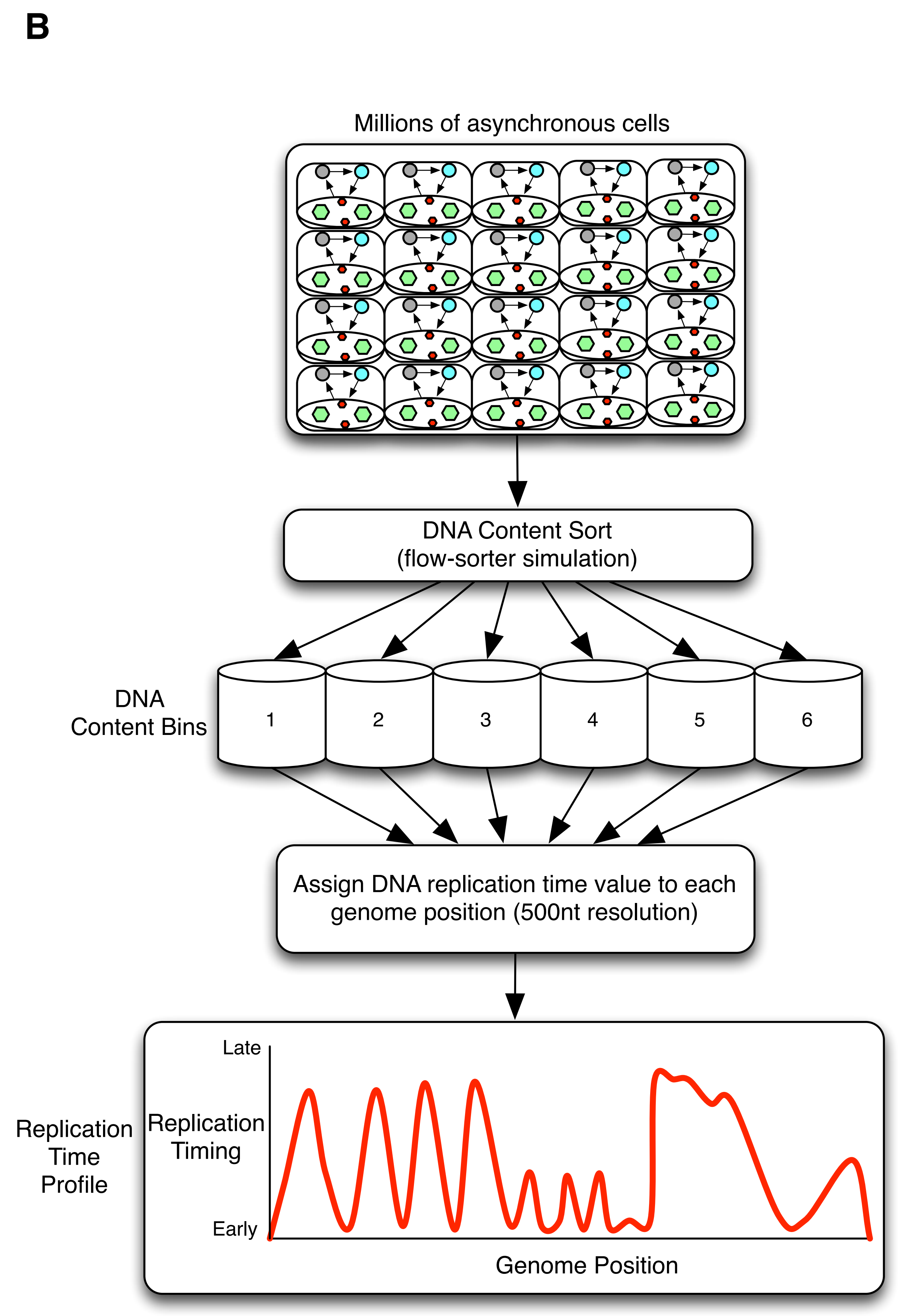

Figure S1

Overview of DNA replication model.

(A) Model inputs are the number of replication forks and the IPLS to specify the probability of initiation of replication at given positions in the genome. The model transitions from G state (resting) to S state (replicating) at random. Once in the S state, the state of the replication forks are queried in an arbitrary sequential order. If the fork is not engaged, the model selects a random un-replicated position on the chromosome and initiates replication at that position with a probability that is assigned by the IPLS for that position. If DNA replication is initiated, then two replication forks are engaged and assigned to move in opposite directions. All forks move one step at a time, disengaging when they collide or reach the chromosome boundary. Model's progress is recorded periodically. Model exits the S state when all DNA is replicated. **(B)** The simulation consists of millions of asynchronous cells. In order determine the DNA replication timing profile, the cell population is first separated according to DNA content into one of six bins. DNA replication time is calculated for each genomic position (500bp resolution) by taking the average of the product of DNA content bin number and the number of occurrences of the genome coordinate in each bin. For visualization, DNA replication timing is plotted as a function of genome position.
