# Supplementary material for: A chromatin structure‐based model accurately predicts DNA replication timing in human cells
Source: Mol Syst Biol. 2014 Mar 28;10(3):722. doi: 10.1002/msb.134859 (PMC4017678; doi:10.1002/msb.134859)
Supplement: Supplementary file 2 — Supplementary Figure S2 [file MSB-10-3-722-s03.pdf]

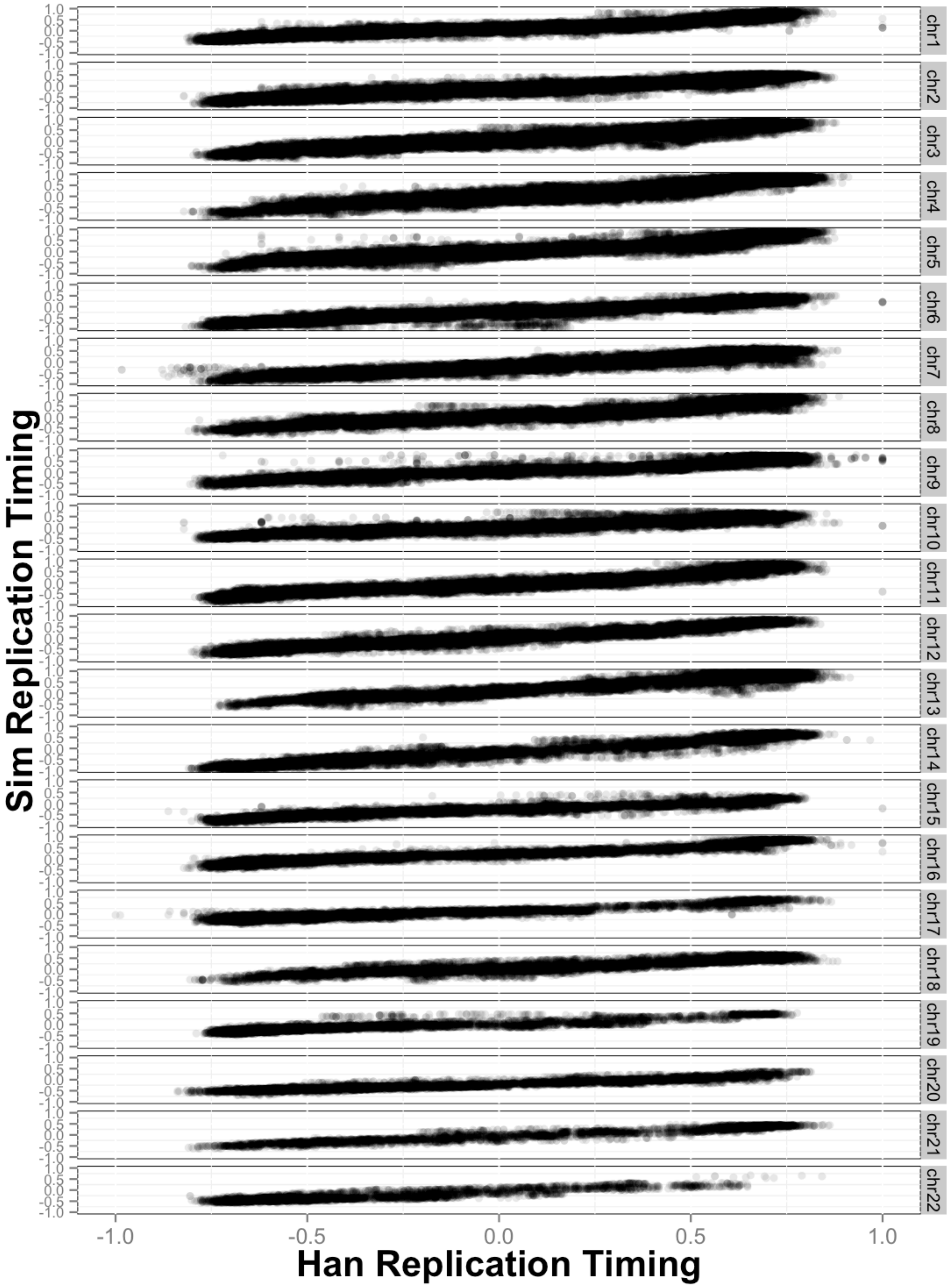

Figure S2

Simulated and empirical DNA replication timing are highly correlated.

Each point represents a replication time assignment for a 500 nt bin for 22 autosomal chromosomes of GM06990 cells. Simulated replication timing assignment is given on the y-axis and the experimentally derived assignment is given on the x-axis. Contour overlay is meant to aid in interpreting plot density.
