# Supplementary material for: A chromatin structure‐based model accurately predicts DNA replication timing in human cells
Source: Mol Syst Biol. 2014 Mar 28;10(3):722. doi: 10.1002/msb.134859 (PMC4017678; doi:10.1002/msb.134859)
Supplement: Supplementary file 3 — Supplementary Figure S3 [file MSB-10-3-722-s05.pdf]

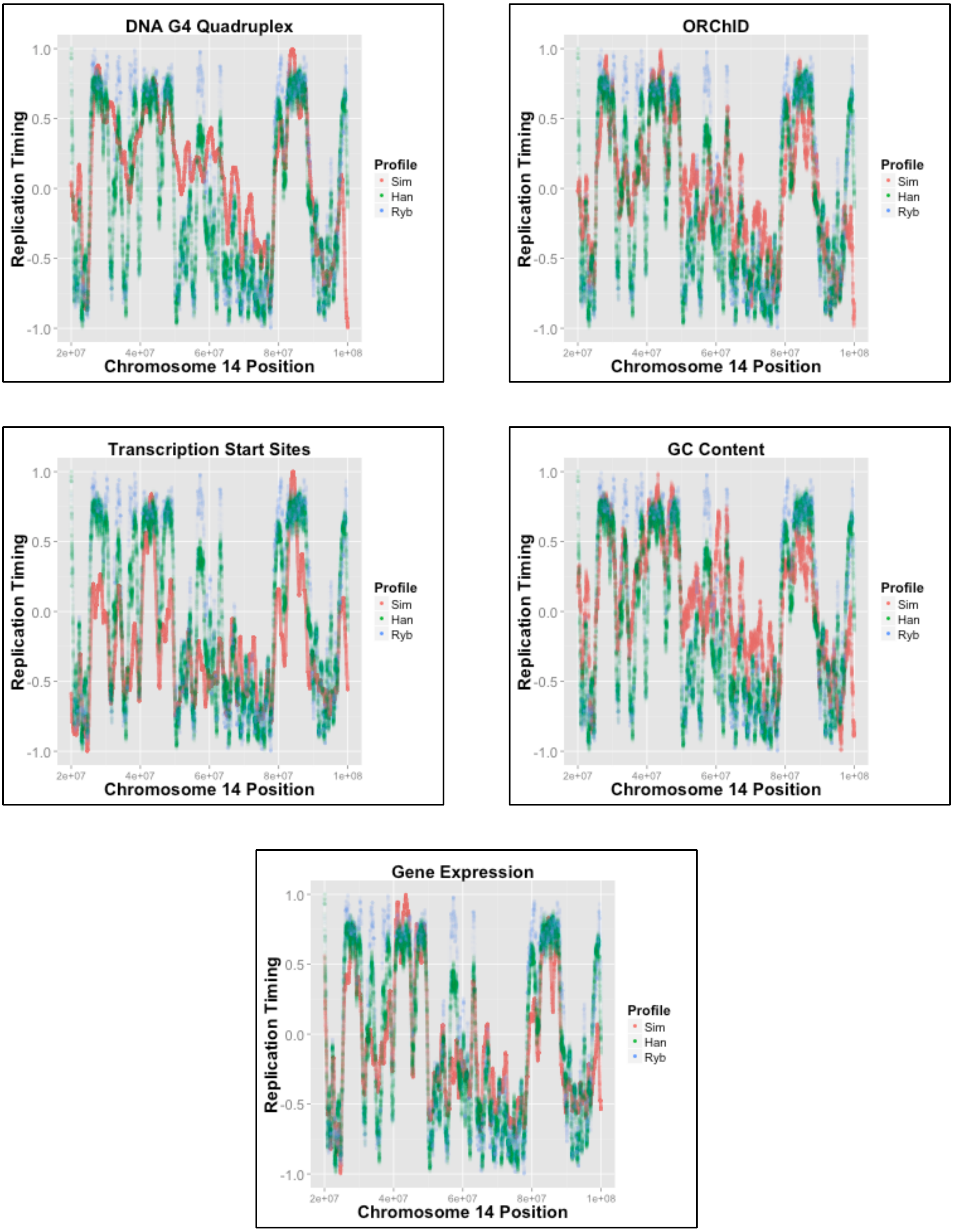

Figure S3

Experimental and simulated timing on chromosome 14 in GM06990 for sequence features and gene expression. Simulations based on genome sequence features (GC content, CpG islands), on local genome conformation (ORChID, G-quadruplex), and on organism-wide transcription start sites generate timing patterns that correlate well with replication timing in five cell lines. Simulated profiles (red) are plotted on the same axes as two empirical datasets: the Hansen (Han) dataset (red) and the Ryba (Ryb) dataset (blue).
