# Supplementary material for: A chromatin structure‐based model accurately predicts DNA replication timing in human cells
Source: Mol Syst Biol. 2014 Mar 28;10(3):722. doi: 10.1002/msb.134859 (PMC4017678; doi:10.1002/msb.134859)
Supplement: Supplementary file 4 — Supplementary Figure S4 [file MSB-10-3-722-s07.pdf]

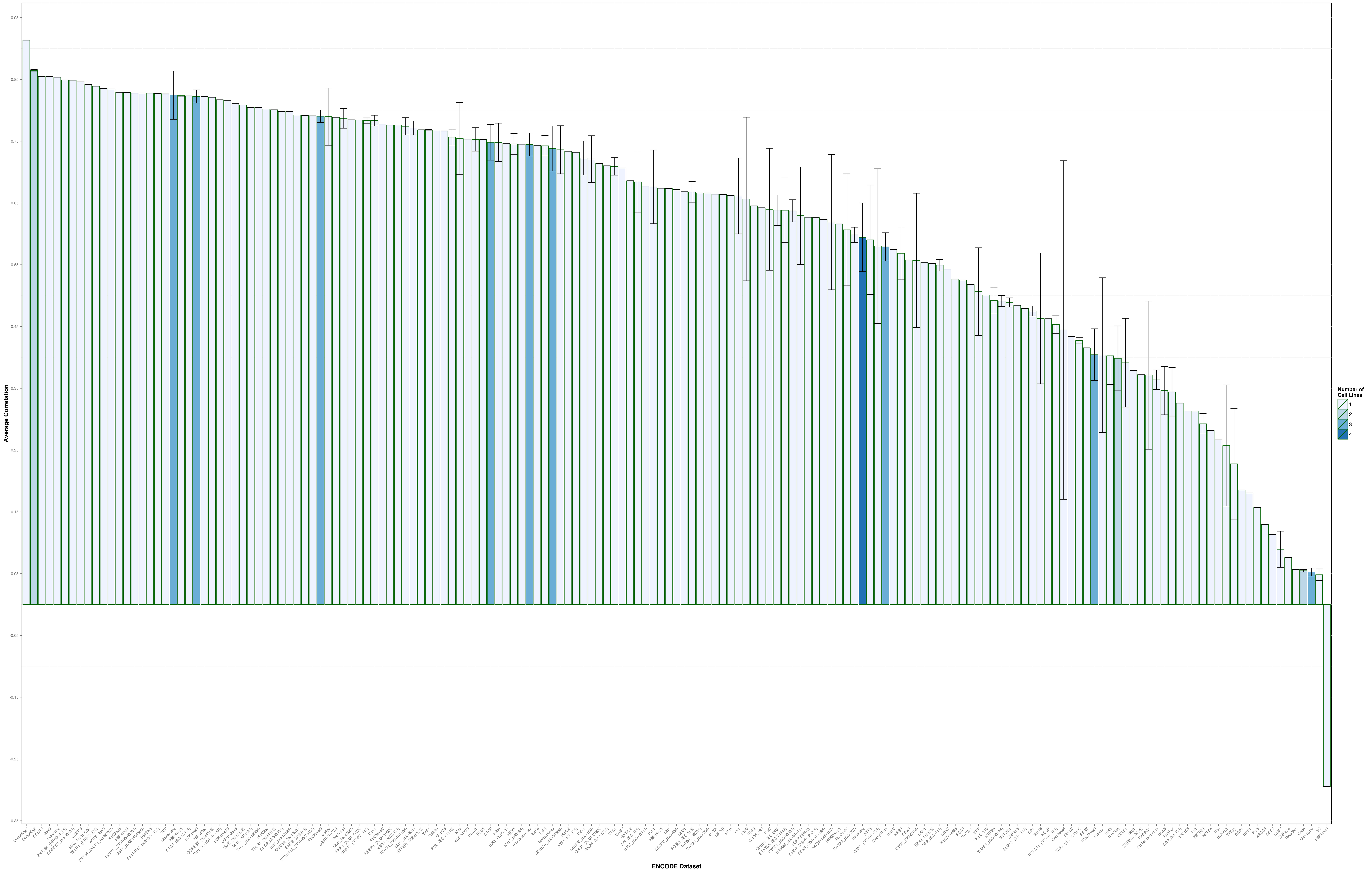

Figure S4

Correlations between simulated and experimental replication timing depends on the genomic mark used in the simulation. DNA replication timing was simulated based on all ENCODE datasets available in at least one of the four cell lines (BG02, BJ, GM06990, K562) for which we had empirical timing data. Subsequently the predicted timing was correlated to the empirical data-set in the matching cell. Error bars represent the standard error of the mean when ENCODE annotations were available for at least two experiments. The shade of the bars signifies the number of cells with the corresponding annotation. For top ranking annotations, DNase DGF, additional optimizations were performed in order to adjust gating-parameters of the simulated flow-sorter to the unknown values used when generating the experimental timing data (see supplemental material). The results are denoted as DNase Dgf*.
