# Supplementary material for: A chromatin structure‐based model accurately predicts DNA replication timing in human cells
Source: Mol Syst Biol. 2014 Mar 28;10(3):722. doi: 10.1002/msb.134859 (PMC4017678; doi:10.1002/msb.134859)
Supplement: Supplementary file 5 — Supplementary Figure S5 [file MSB-10-3-722-s09.pdf]

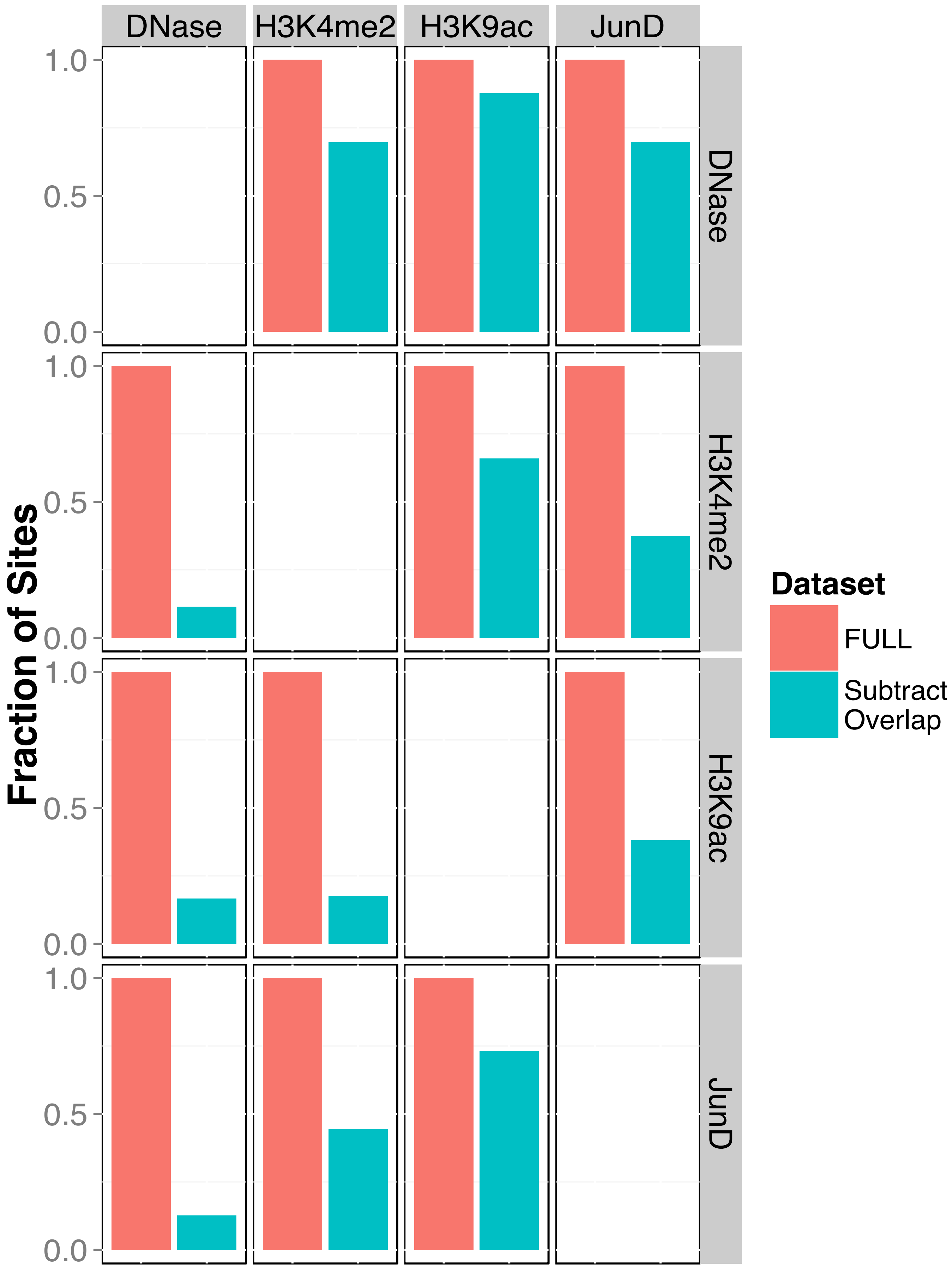

Figure S5

The extent of genome localization overlap between top DNA replication timing-predicting genomic marks.

Using the datasets presented in Figure 2b, rows indicate the dataset and columns indicate the effect of subtracting a dataset. Each vertical bar represents the fraction of genomic regions in the dataset: original dataset (red) and reduced dataset (blue).
