# Supplementary material for: A chromatin structure‐based model accurately predicts DNA replication timing in human cells
Source: Mol Syst Biol. 2014 Mar 28;10(3):722. doi: 10.1002/msb.134859 (PMC4017678; doi:10.1002/msb.134859)
Supplement: Supplementary file 6 — Supplementary Figure S6 [file MSB-10-3-722-s11.pdf]

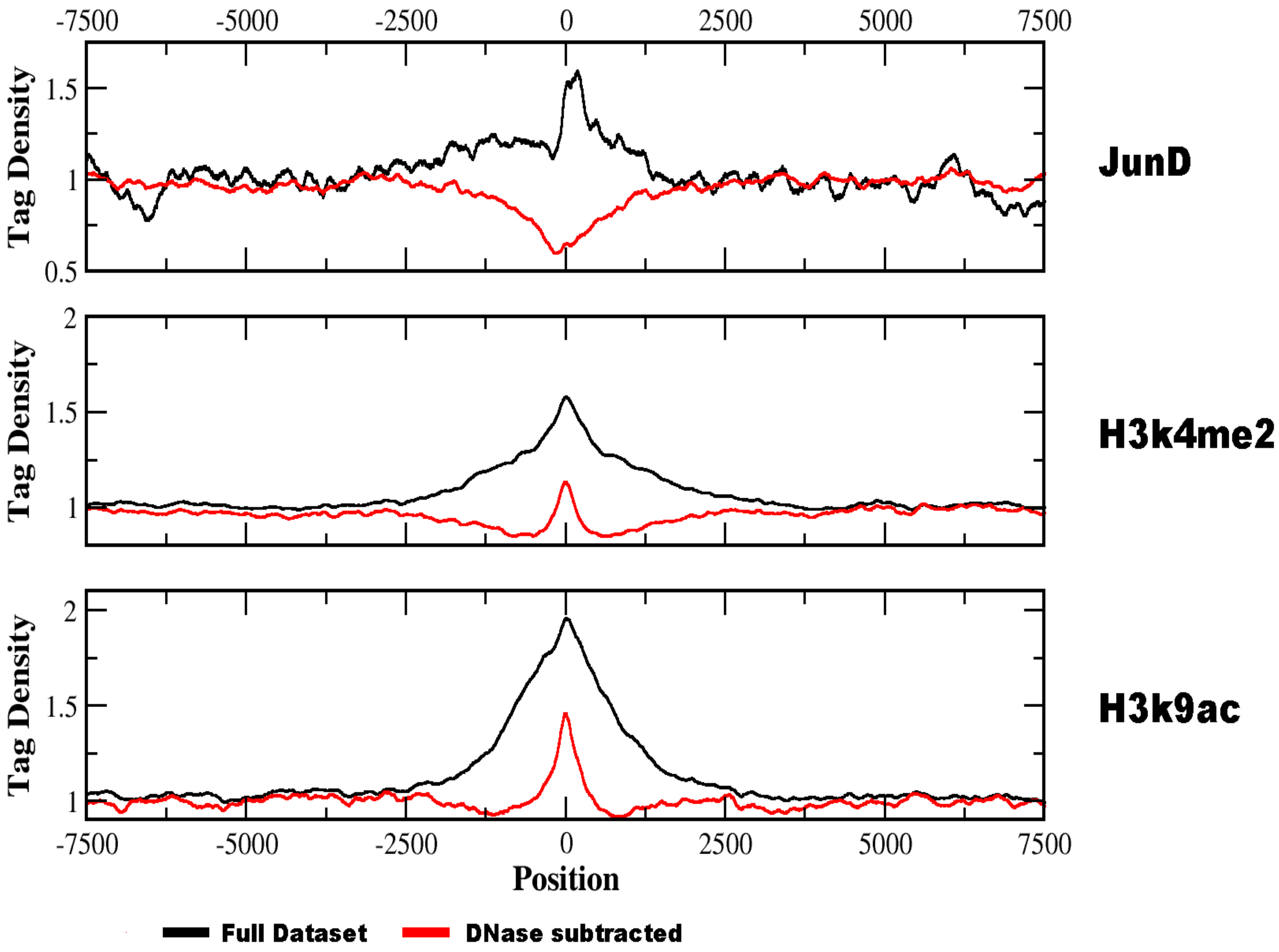

Figure S6

Replication initiation less likely to occur at ENCODE marks not overlapping DNASE sites.

The tag density of initiation sequencing reads around JunD, H3k4me2 and H3k9ac sites are much lower for sites not overlapping DNase sites (red) than those that do (black curve). Plots are normalized to 1 at large distances. A value below 1, observed in the vicinity of all non-DNase overlapping marks, indicates *suppression* of replication initiation compared to the "average" genomic location.
