# Supplementary material for: A chromatin structure‐based model accurately predicts DNA replication timing in human cells
Source: Mol Syst Biol. 2014 Mar 28;10(3):722. doi: 10.1002/msb.134859 (PMC4017678; doi:10.1002/msb.134859)
Supplement: Supplementary file 7 — Supplementary Figure S7 [file MSB-10-3-722-s13.pdf]

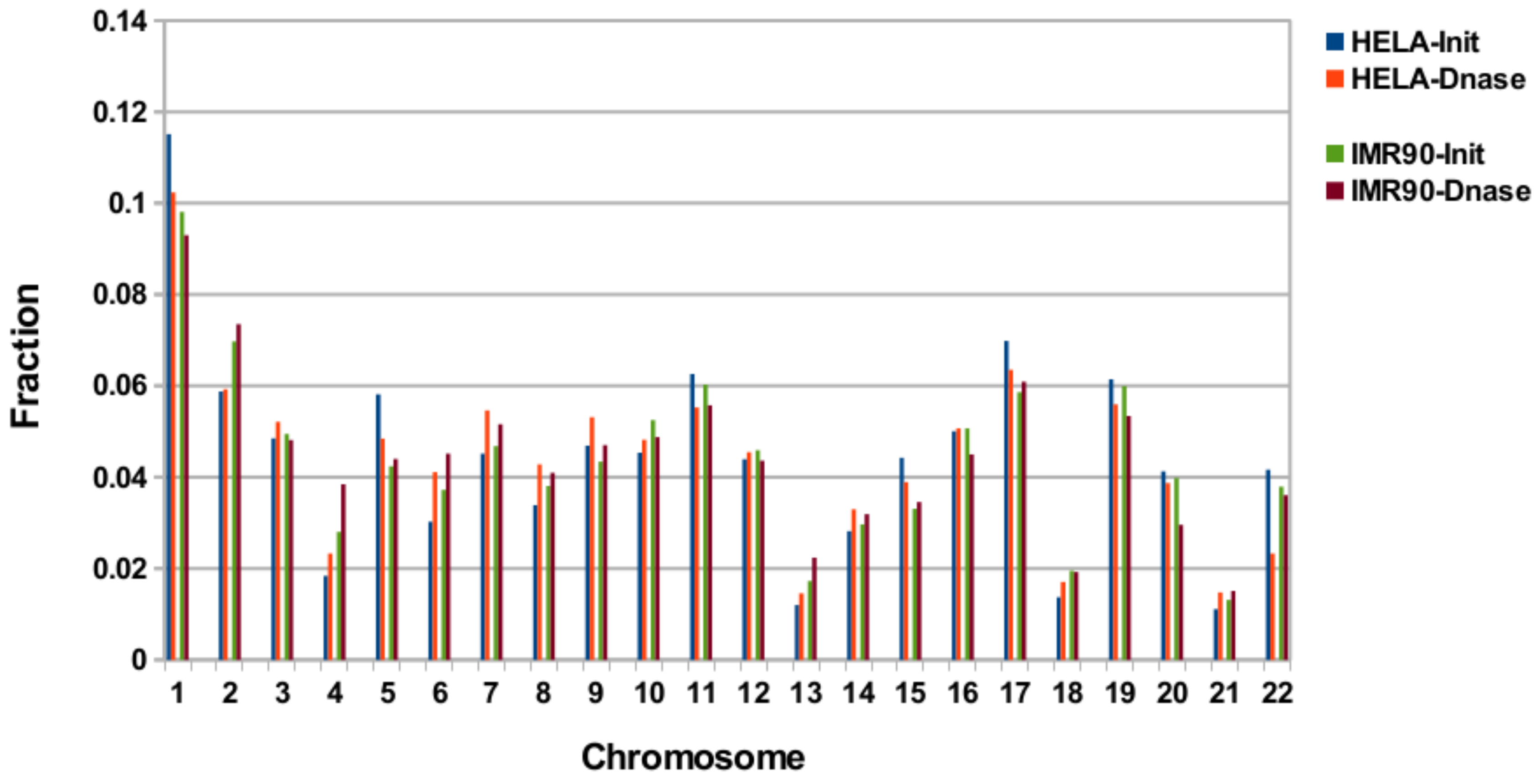

Figure S7

The distribution of DNase HS sites across chromosomes closely follows the number of initiation sites.

Shown is the fraction of the total number of DNase hypersensitive sites and replication initiation peaks (derived from (Besnard et al., 2012)) across all autosomal chromosomes.
