# Supplementary material for: A chromatin structure‐based model accurately predicts DNA replication timing in human cells
Source: Mol Syst Biol. 2014 Mar 28;10(3):722. doi: 10.1002/msb.134859 (PMC4017678; doi:10.1002/msb.134859)
Supplement: Supplementary file 8 — Supplementary Figure S8 [file MSB-10-3-722-s15.pdf]

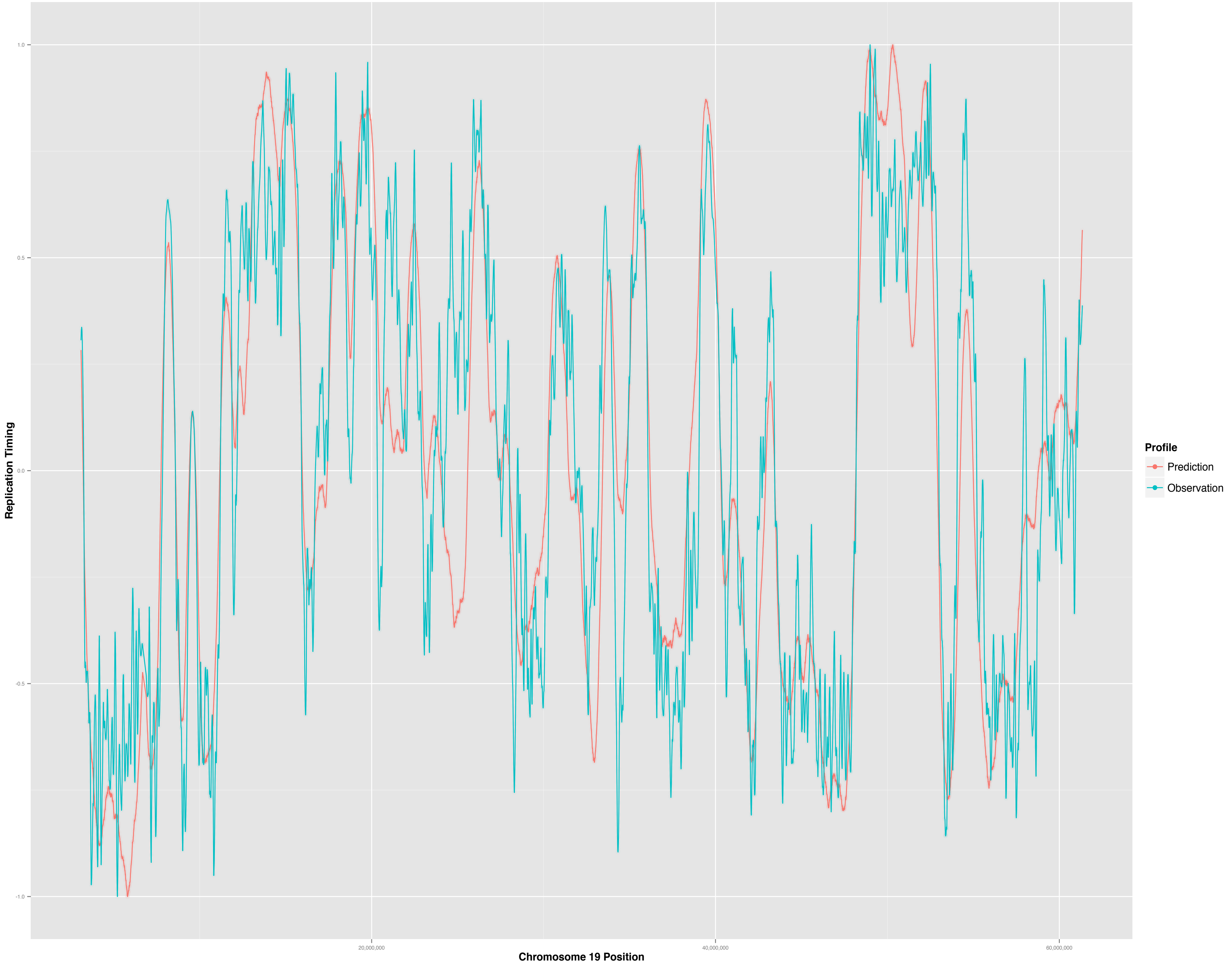

Figure S8

DNA replication timing predictions applied to mouse cells. Plotted are simulated (red) and observed (blue) DNA replication timing (y-axis) profiles for chromosome 19 (x-axis) of mouse embryonic fibroblast cells. Simulated profile is based on DNase HS data for NIH/3T3 cells. Observed data is derived from MEF cells.
