# Supplementary material for: A chromatin structure‐based model accurately predicts DNA replication timing in human cells
Source: Mol Syst Biol. 2014 Mar 28;10(3):722. doi: 10.1002/msb.134859 (PMC4017678; doi:10.1002/msb.134859)
Supplement: Supplementary file 9 — Supplementary Figure S9 [file MSB-10-3-722-s17.pdf]

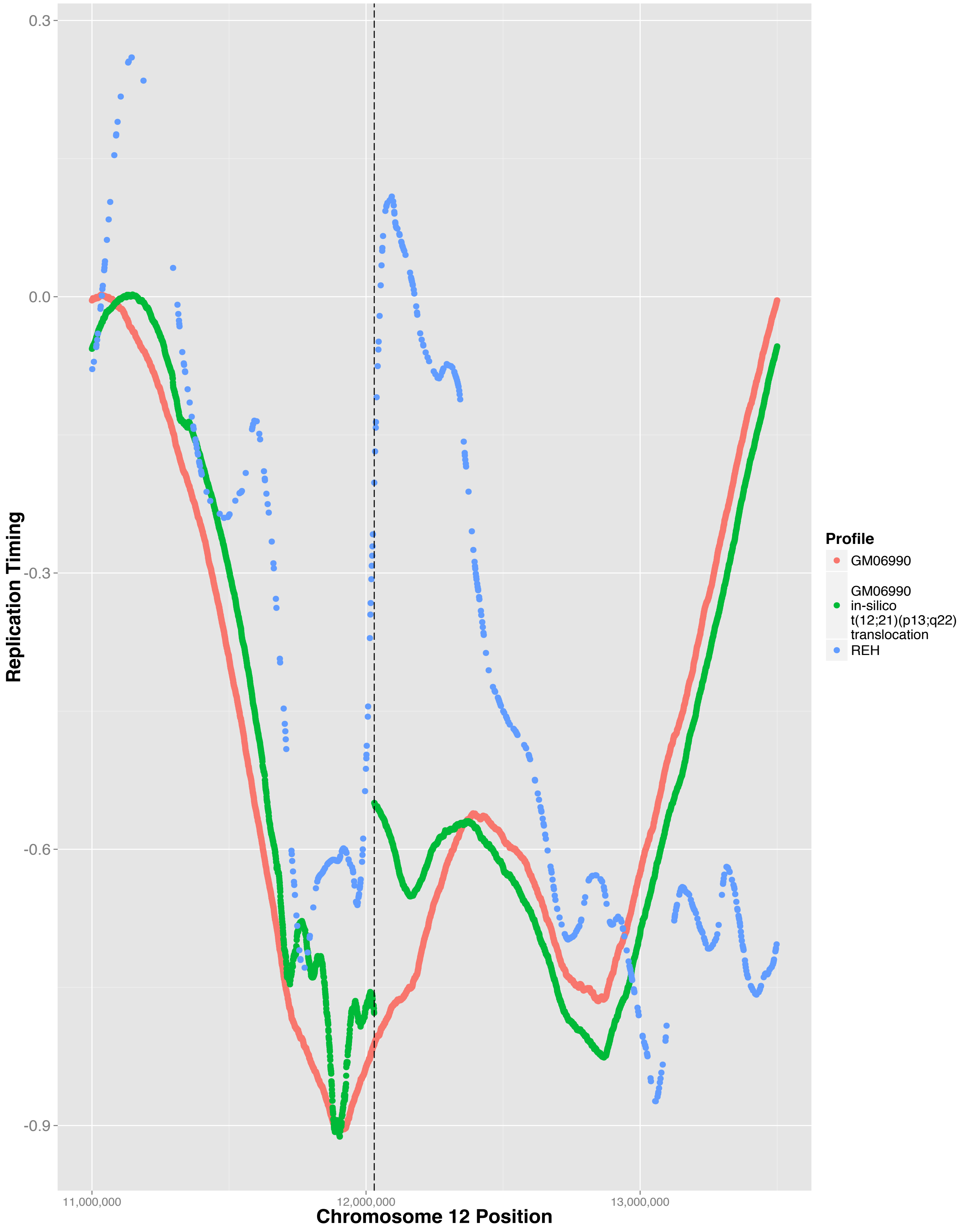


# Figure S9

**A translocation event simulated *in silco* in GM06990 cells qualitatively reproduces the timing discontinuity observed** (Wiemels et al., 2000) **at a TEL-AML1 translocation in ALL**. Simulated replication profile of *in silico* translocated (green line) normal (red line) are plotted on the same set of coordinates. Also shown is the experimentally observed replication timing profile of REH cells, which harbor the translocation (blue dotted line). The REH and GM06990 *in silico* translocated profiles show an abrupt change in replication timing from early to late at the breakpoint site (dotted vertical line).
