# Supplementary material for: A chromatin structure‐based model accurately predicts DNA replication timing in human cells
Source: Mol Syst Biol. 2014 Mar 28;10(3):722. doi: 10.1002/msb.134859 (PMC4017678; doi:10.1002/msb.134859)
Supplement: Supplementary file 10 — Supplementary Figure S10 [file MSB-10-3-722-s19.pdf]

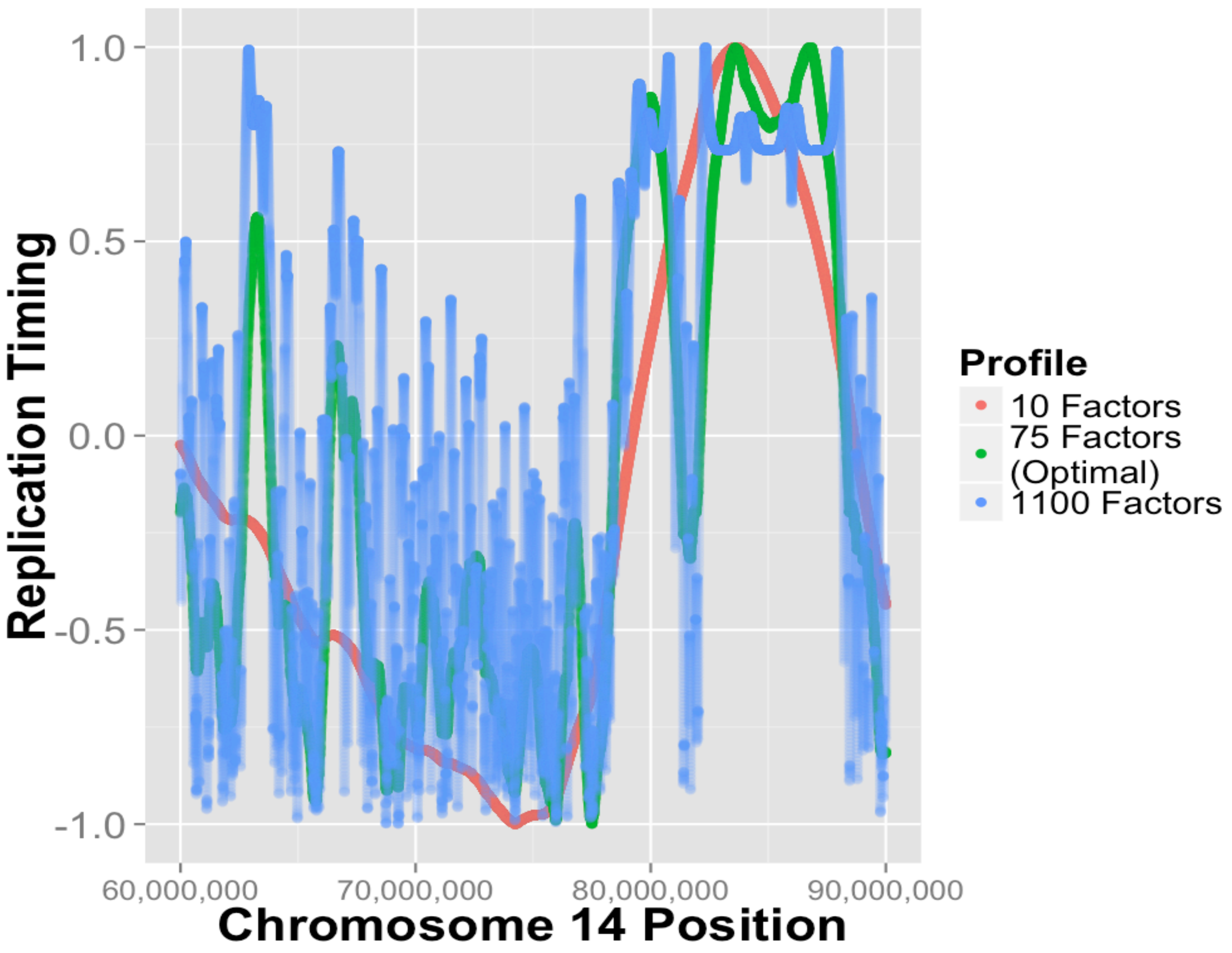

Figure S10

Predicted DNA replication profile depends on the number of DNA replication forks. DNA replication timing was simulated for GM06990 cells based on DNase data using a range of the maximal number *N* of replication 'factors' for chromosome 14. Simulations with few (10 factors; red profile) produce smooth DNA replication timing profiles, owing to the long distance that each factor travels. Simulations with many (1100 factors; blue profile) produce rough landscapes owing to the short distance that each factor travels. The number of replication factors that produce
