# Supplementary material for: A chromatin structure‐based model accurately predicts DNA replication timing in human cells
Source: Mol Syst Biol. 2014 Mar 28;10(3):722. doi: 10.1002/msb.134859 (PMC4017678; doi:10.1002/msb.134859)
Supplement: Supplementary file 11 — Supplementary Figure S11 [file MSB-10-3-722-s21.pdf]

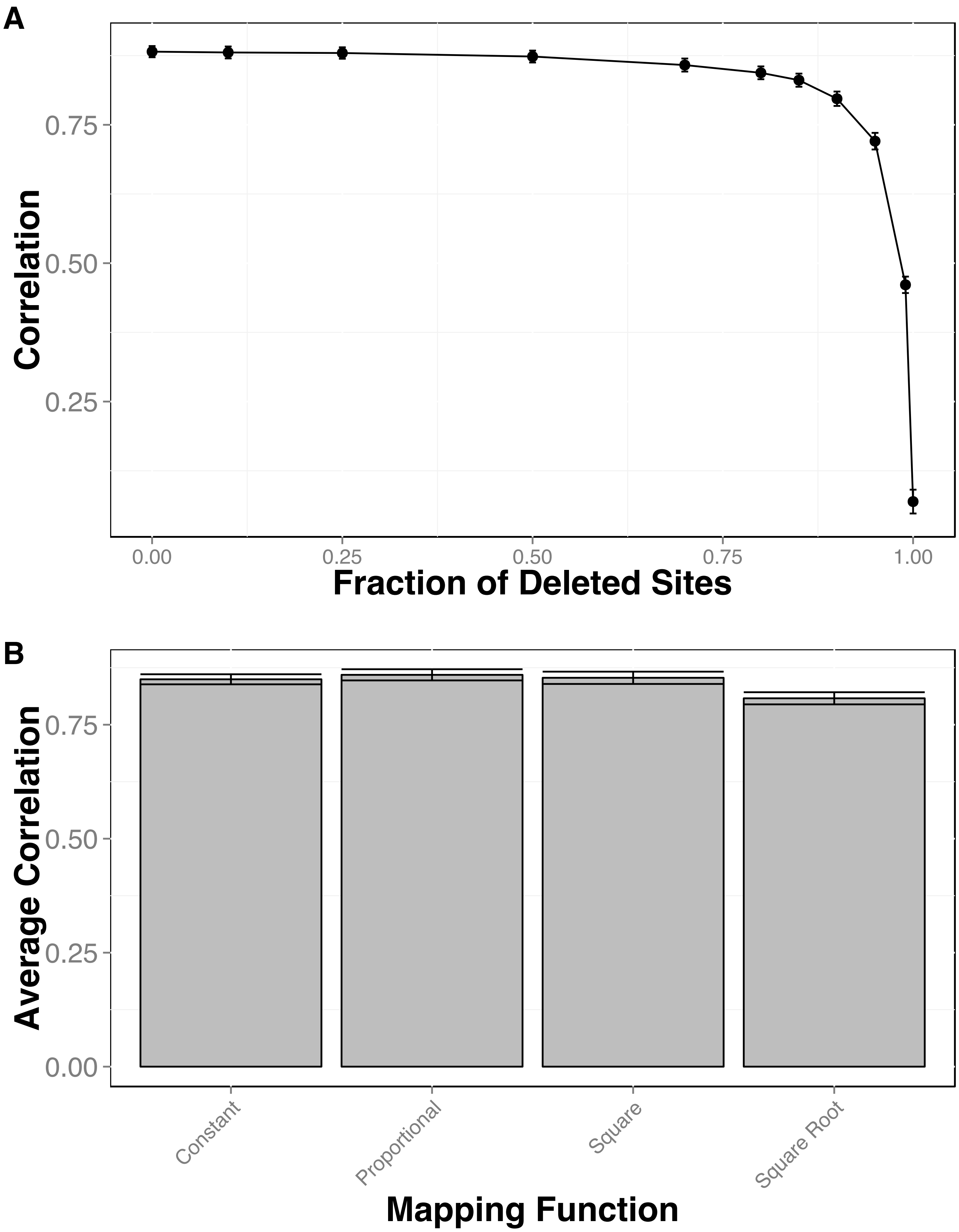

Figure S11

Mechanistic model is highly robust to simulation parameters. (A) Randomly eliminating an ever larger fraction (x-axis) of DNase HS marks from the simulation of replication timing in GM06900 cells reduces the correlation (y-axis) of the model prediction with the empirical data only marginally when removing up to 50% of DNase sites. Beyond that, point, the correlation degrades gradually. Error bars represent standard error of the mean resulting from averaging the correlation value across 22 autosomal chromosomes. **(B)** DNA replication timing was predicted for K562 and GM06990 cells based on cell-specific DNase data. Different functions were used to mapping the amplitudes ***a(x)*** of the DNase signal at a location ***x*** in the ENCODE database to the probability ***p(x)*** of replication initiation at this DNase mark. No significant differences were seen between assigning a constant value ***p*** = 1 or amplitude dependent values $p(x) \sim a(x)$, $p(x) \sim a(x)^2$, $p \sim a(x)^{1/2}$.
