# Supplementary material for: A chromatin structure‐based model accurately predicts DNA replication timing in human cells
Source: Mol Syst Biol. 2014 Mar 28;10(3):722. doi: 10.1002/msb.134859 (PMC4017678; doi:10.1002/msb.134859)
Supplement: Supplementary file 12 — Supplementary Figure S12 [file MSB-10-3-722-s23.pdf]

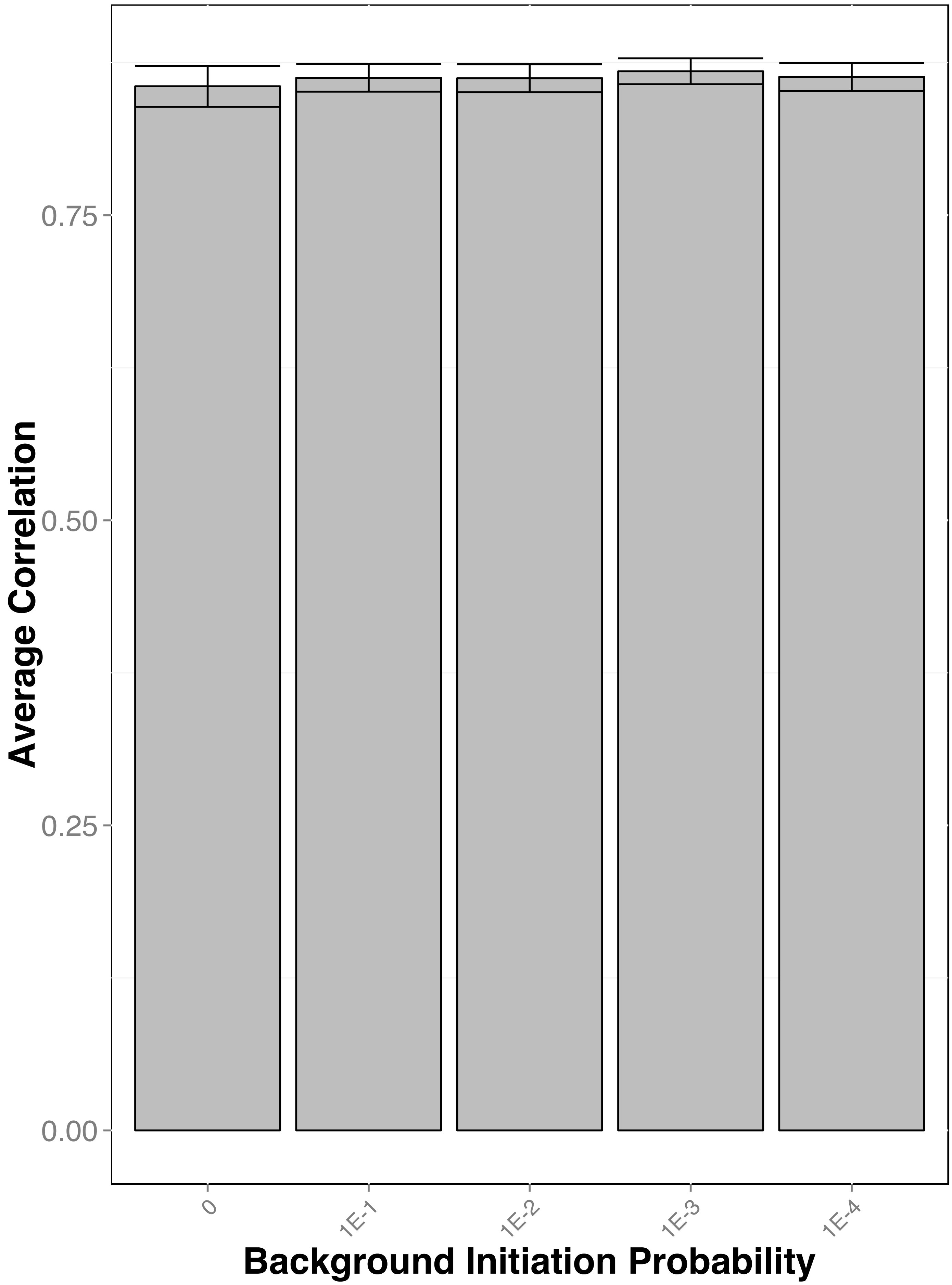

Figure S12

Background initiation probability does not affect the accuracy of model's predictions. DNA replication timing was predicted using DNase HS IPLS model as input for a range of background initiation probabilities (x-axis). In each case the average correlation with empirical data (y-axis) is only marginally affected. Error bars are standard error of the mean arising from averaging correlations across 22 autosomal chromosomes.
