# Supplementary material for: A chromatin structure‐based model accurately predicts DNA replication timing in human cells
Source: Mol Syst Biol. 2014 Mar 28;10(3):722. doi: 10.1002/msb.134859 (PMC4017678; doi:10.1002/msb.134859)
Supplement: Supplementary file 13 — Supplementary Figure S13 [file MSB-10-3-722-s25.pdf]

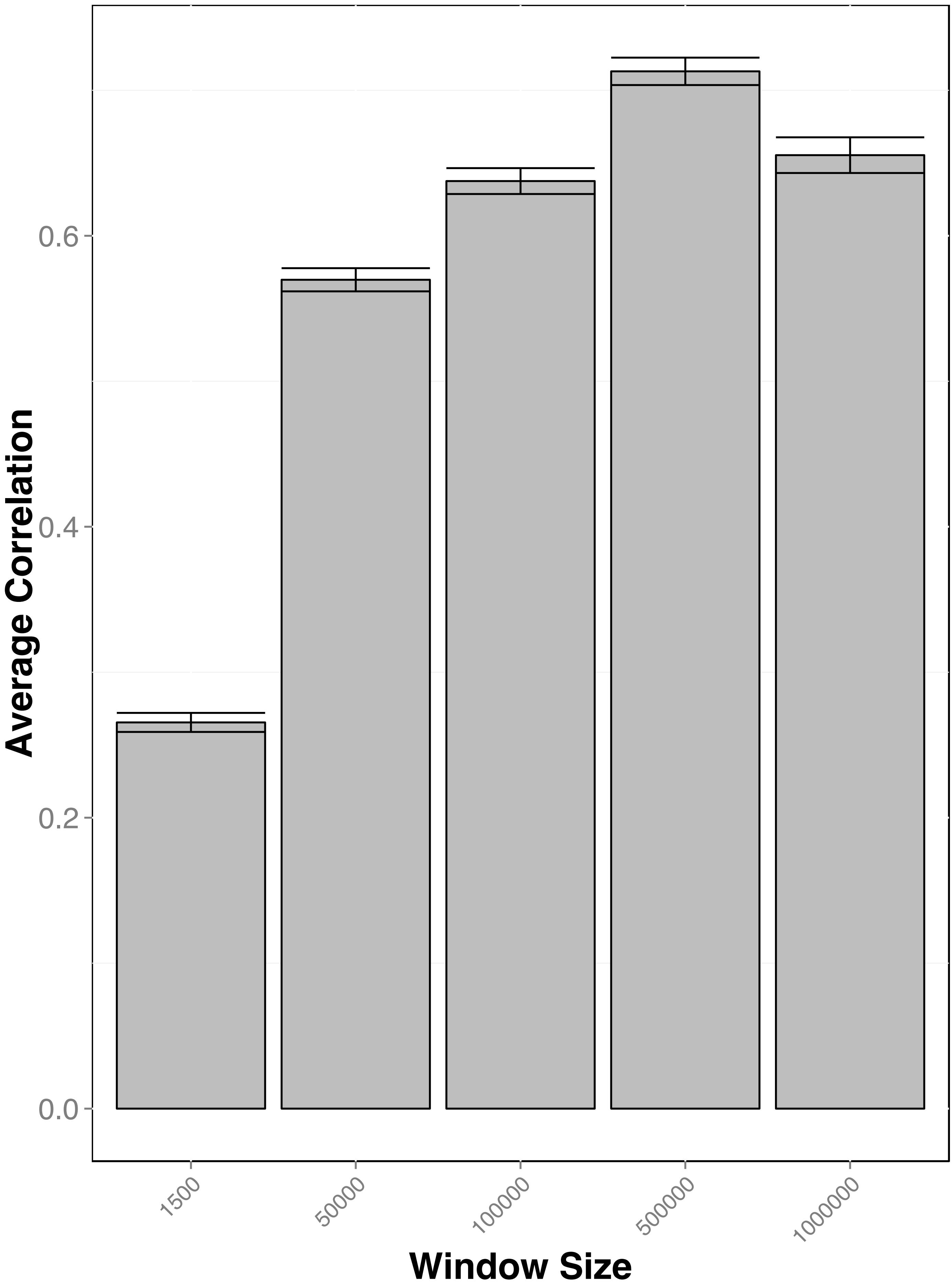

Figure S13

DNase HS site density correlates with DNA replication timing. The number of DNase HS were summed for various sliding genome widows (x-axis) and correlated with empirical DNA replication timing data for same cells (GM06990). The best correlation (r=0.71) was obtained using the 0.5MB window. Error bars represent the standard error of the mean resulting from averaging correlation across 22 autosomal chromosomes.
