# Supplementary material for: A chromatin structure‐based model accurately predicts DNA replication timing in human cells
Source: Mol Syst Biol. 2014 Mar 28;10(3):722. doi: 10.1002/msb.134859 (PMC4017678; doi:10.1002/msb.134859)
Supplement: Supplementary file 14 — Supplementary Figure S14 [file MSB-10-3-722-s27.pdf]

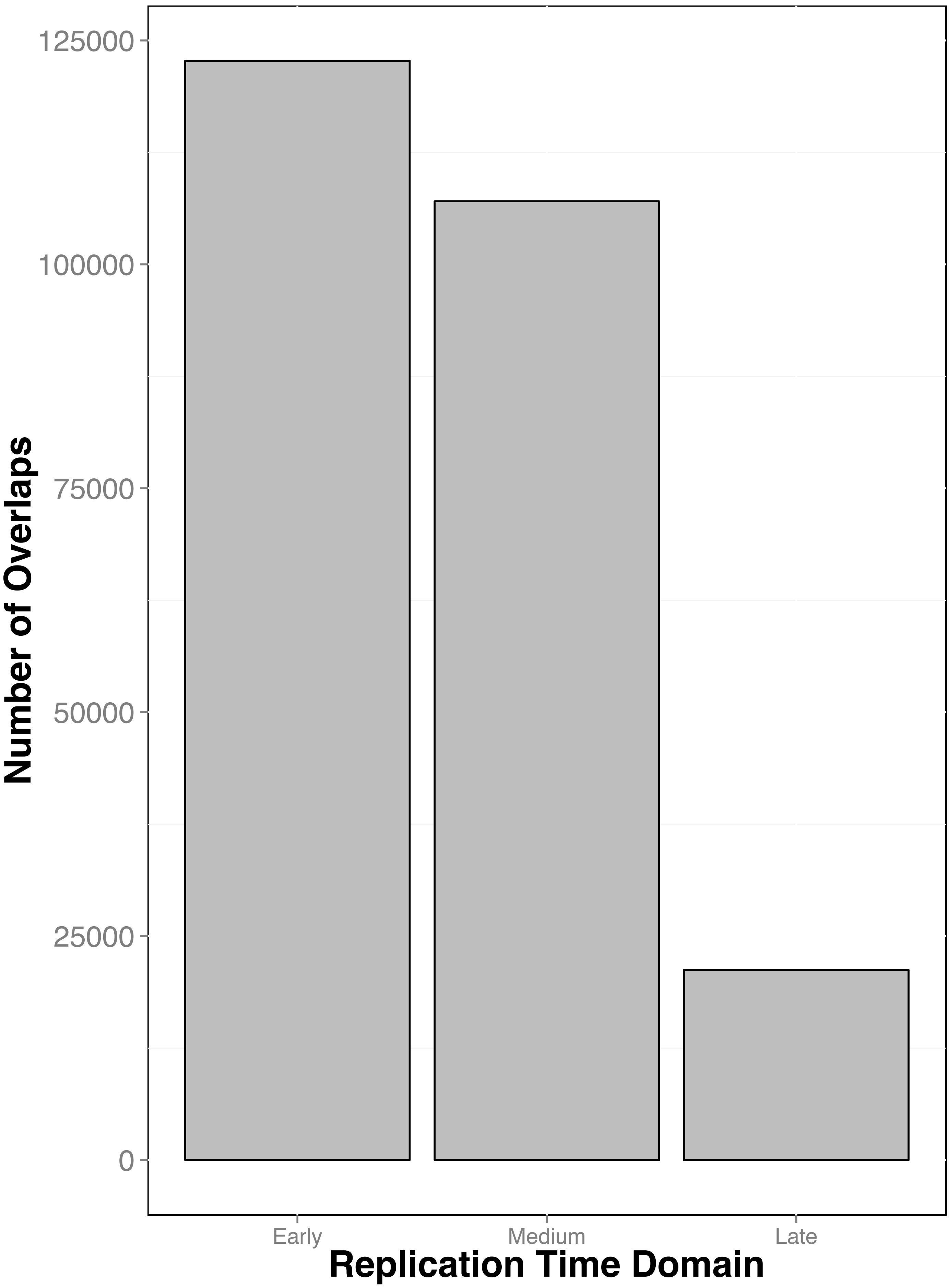

Figure S14

Majority of DNase HS sites reside in early or medium replication timing domains. DNase HS were assigned into one of three DNA replication timing domain bins (x-axis) by consulting empirical data for matching cells (GM06990). The number of DNase sites in each bin were then counted (y-axis).
