# Supplementary material for: A chromatin structure‐based model accurately predicts DNA replication timing in human cells
Source: Mol Syst Biol. 2014 Mar 28;10(3):722. doi: 10.1002/msb.134859 (PMC4017678; doi:10.1002/msb.134859)
Supplement: Supplementary file 15 — Supplementary Figure S15 [file MSB-10-3-722-s29.pdf]

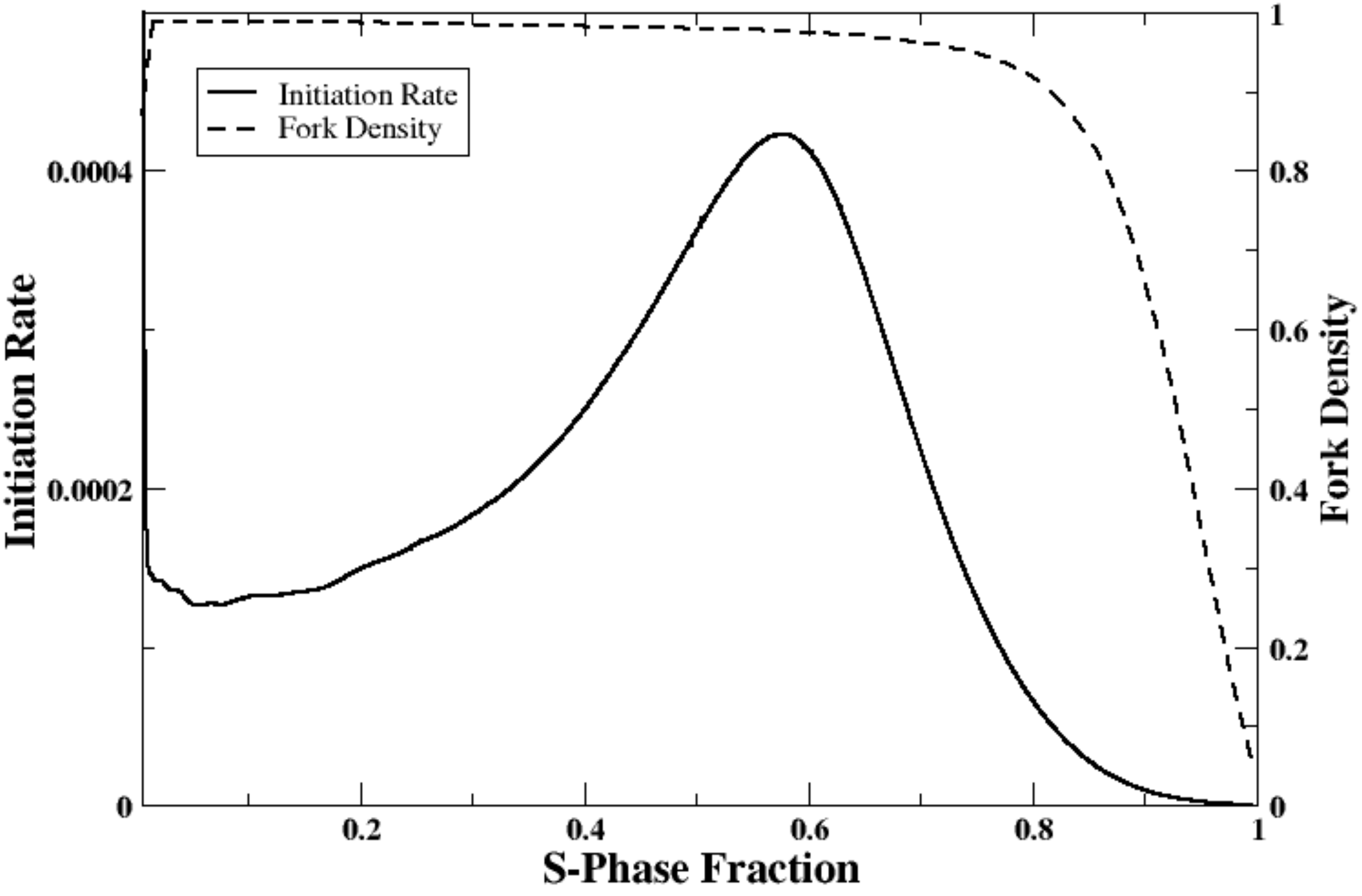

Figure S15

Change in global initiation rate and fork density over S-phase. The initiation rate (number of initiation events per kilobase) and fork density (fraction of engaged replication forks) were averaged for simulated cell population and plotted as a function of S-phase fraction (time along the S-phase).
