# Supplementary material for: A chromatin structure‐based model accurately predicts DNA replication timing in human cells
Source: Mol Syst Biol. 2014 Mar 28;10(3):722. doi: 10.1002/msb.134859 (PMC4017678; doi:10.1002/msb.134859)
Supplement: Supplementary file 18 — Supplementary Information [file MSB-10-3-722-s33.pdf]

SUPPLEMENTARY INFORMATION S1

Background replication initiation probability does not affect simulation results

In addition to the high efficiency initiation sites, all IPLSs used in the current work contain a low probability (P = 1E-4) replication initiation background. This step was thought to be necessary to avoid the "random completion" problem (i.e. to ensure complete replication in a finite time). Towards the end of our study we found that this step is, in fact, not required: setting this background probability to zero did not, overall, affect the shape of the timing prediction and lead to a slight increase of the predicted length of the S-phase from 8.3h to 8.7h. Other variants with a non-zero background probabilities did not affect the accuracy of the model predictions: assigning P=0.001, 0.01 and 0.1 to the background did not significantly change the results (**Figure S11B**).

Overlap with empirical initiation confirm that DNase HS are the main determinants of DNA replication timing

We show that most DNA replication timing-predictive ENCODE marks did not maintain a high level of precision when marks overlapping with DNase HS sites were removed. From this result, it is reasonable to expect to see differences in how strongly the complete and the DNase subtracted sets overlap with empirically determined replication *initiation* events. Besnard (Besnard et al., 2012) and co-workers determined the global probability of replication initiation events by large-scale sequencing of nascent DNA. The read-tag density for these data show (**Figure S6**) clearly discernible maxima at the location of H3k9ac, H3k4me2 and JunD genomic marks. However, after removing marks overlapping with DNase HS sites, the signal is significantly diminished. Moreover, there is evidence of a weak suppression of initiation events at the remaining genome marks. These results reinforce our findings that DNase HS sites are the main independent determinants of DNA replication timing.

Simulated flow-sorter optimization improves prediction accuracy

In the laboratory, replication timing is observed by BrdU labeling of un-synchronized cells followed by flow-sorting with respect to DNA content in order to assign a cell-cycle time (Ryba et al., 2011) to each cell. In one such work, Hansen and co-workers (Hansen et al., 2010) used 6 pre-defined time-slices termed G_1, S_1, S_2, S_3, S_4, G_2 (in increasing temporal order) separated by five adjustable gates (in addition to 0 and 1) during flow sorting. Naturally, these parameters, as well as the response curve of the flow-sorter, impact the estimate of the average replication times. In order to reduce the non-biological influence of the choice for gating parameters, we performed a simulated annealing optimization of flow-sorter gate settings for the top predictive model based on DNase HS sites. It improved the average correlation between experiment and simulation by 3% (from r=0.89 to r=0.92). At this level, our prediction approaches the reproducibility levels between experiments: we found that timing measurements for same cells line (GM06990) performed in different laboratories (Hansen (Hansen et al., 2010) and Gilbert (Ryba et al., 2012) datasets, respectively) were correlated at a level r=0.94, only marginally better than our best model prediction.

Simplified DNase HS density based model produces less accurate predictions

One may wonder if it is possible to built a simpler model based on the local density of DNase HS sites alone, rather than using the more complex diffusion/collision model presented in this work (**Figures 1A and S1**). DNase HS have been associated (Aladjem, 2007) with early replication in several earlier studies (for a review see (Aladjem, 2007)). Re-analysis of DNase HS ENCODE data and experimental replication timing data confirms that more than 90% of DNase HS sites are located in early or medium replication timing domains (**Figure S14**). However, when trying to utilize this information to built a predictive quantitative replication timing model, one also has to answer the question of the timing for regions without DNase sites, in particular how far does the “early” timing information propagate

around DNase sites? In our diffusion model (**Figures 1A and S1**) this question is answered dynamically by collision with other replication forks. A simplified model might use a fixed-size window instead, assigning a timing to each location depending on the number and intensity of DNase HS sites around the location. We simulated this model and systematically tried several fixed window-sizes (**Figure S13**). While this simple model correlates well with the experimental timing data, the correlation never exceeds 0.71, which is significantly lower compared to when the same data are input into the mechanistic model.
