# Supplementary material for: A chromatin structure‐based model accurately predicts DNA replication timing in human cells
Source: Mol Syst Biol. 2014 Mar 28;10(3):722. doi: 10.1002/msb.134859 (PMC4017678; doi:10.1002/msb.134859)
Supplement: Supplementary file 19 — Replicon software package [file MSB-10-3-722-s34.gz › replicon/README.pdf]

Replicon

February 10, 2014

Contents

1 Prerequisites **1**

2 Introduction **1**
2.1 What's inside the example directory 2

3 Obtaining a BED-formatted file of genomic annotations **3**
3.1 Downloading a BED from Table Browser 3

4 Generating an IPLS **3**
4.1 BedToIPLS options 4

5 Predicting and visualizing replication timing **5**

6 Replicon options **7**

1 Prerequisites

1. Compiled replicon binary (see 'INSTALL.txt')
2. R is required for generating plots
3. About 7GB of available RAM
4. Steps in this document assumes that your current working directory is 'example' and that the 'replicon' executable is in the parent directory.

2 Introduction

The goal of this document is to allow an interested reader to replicate findings presented in the manuscript. To that end, we provide the source code for Replicon – the software that was used to simulate replication timing. We also provide two accessory tools: RepliconWrench to convert a BED-formatted file

to an IPLS file as well as a Perl script to convert Replicon's output file to an easy-to-interpret format suitable for creating visual plots.

The 'example' directory contains IPLS files (under the 'ipls' directory) and replication timing prediction files (under the 'replicationTiming' directory). Please note that executing the 'runExample.sh' script will re-generate the directories and files. See below for additional details and step-by-step instructions outlining how to obtain genome annotation files (see section 3), how to convert them to IPLS files (see section 4) and how to predict replication timing from IPLS files (see section 5). Replicon options are outlined in section 6. You are also encouraged to examine the 'runExample.sh' script to see how all the above steps could be bundled together.

2.1 What's inside the example directory

Description of files contained in 'ipls' and 'replicationTiming' directories:

- The 'ipls' directory contains 22 sub-directories each corresponding to an autosomal chromosome under which there is a chromosome-specific IPLS file. For instance: 'chr1_GM06990' is the chromosome 1 IPLS for GM06990 cells. These files were generated from a set of DNase I HS annotations for GM06990 cells. See section 3 for more information.

- The 'replicationTiming' directory contains replication timing predictions generated from IPLS files [1]. These files come in 3 flavors:
  - '*GM06990.CGHnTiming.csv.gz' – This file relates genomic bins (1st column) to simulated flow-sorter gates. Each successive gate (0 through 5) targets cells with increasing DNA content (see manuscript Methods).
  - '*GM06990.CGHnTiming.csv.timing.gz' – This is a calculation of replication timing for every 500bp bin based on the above file and is somewhat easier to understand (see manuscript Methods). Here, replication time is assigned to every genomic bin with smaller time values corresponding to earlier replication time.
  - '*GM06990.CGHnTiming.csv.timing_plot.png' – This is a line plot of the above file for visualization purposes.

For those readers who are interested in creating their own IPLS files, we provide the following instructions, guiding you trough all the steps required to simulate replication timing using Replicon.

[1] The files with .gz extension have been compressed to save space

3 Obtaining a BED-formatted file of genomic annotations

We include a BED file with this distribution in the 'example' directory. The file, named 'GM06990.bed', is sufficient to generate genome-wide IPLSs (see Section 4). Below we illustrate the steps to create such a file from the set of epigenetic annotations stored at the UCSC Genome Browser directly. These instructions could be expanded to other annotations and are applicable to most BED-formatted files.

3.1 Downloading a BED from Table Browser

The first step is to obtain a set of genome annotations. Here, by way of an example, we will use DNase I HS sites for GM06990 cells. The easiest way to obtain the set of DNase HS sites is from the UCSC Genome Browser by navigating to the Table Browser section, as illustrated in Figure 1. Note the pull-down choices for clade (Mammal), genome (Human), assembly (hg19), group (Regulation), track (UW DNaseI HS), and table (GM06990 PK 1) settings.[2] The next steps:

1. Choose 'BED' from 'output format' menu
2. Provide a file name (we suggest 'GM06990.bed' if you'd to like to follow this manual)
3. Click 'get output'
4. Move the downloaded file to the 'example' directory

Similar procedure would work for any number of annotations either stored at the UCSC genome browser.

4 Generating an IPLS

An IPLS is a two-column, chromosome-specific file where the first column specifies genomic position and the second column specifies the probability of initiating replication at that position. To facilitate generation of IPLS files from BED files, we provide an accessory tool called 'RepliconWrench'. This convenience tool reads a BED file and assigns a probability of initiation to every 500bp bin in a chromosome-specific manner. This probability is scaled according to a 'value' column in the BED file – usually the fourth column, though the column index may be specified using a command line argument (see below). Should a

[2] It may be possible to access the table and settings directly: `http://genome.ucsc.edu/cgi-bin/hgTables?hgsid=362087087&clade=mammal&org=Human&db=hg19&hgta_group=regulation&hgta_track=wgEncodeUwDnase&hgta_table=wgEncodeUwDnaseGm06990PkRep1&hgta_regionType=genome&position=chr21%3A33031597-33041570&hgta_outputType=primaryTable&hgta_outFileName=GM06990.bed`

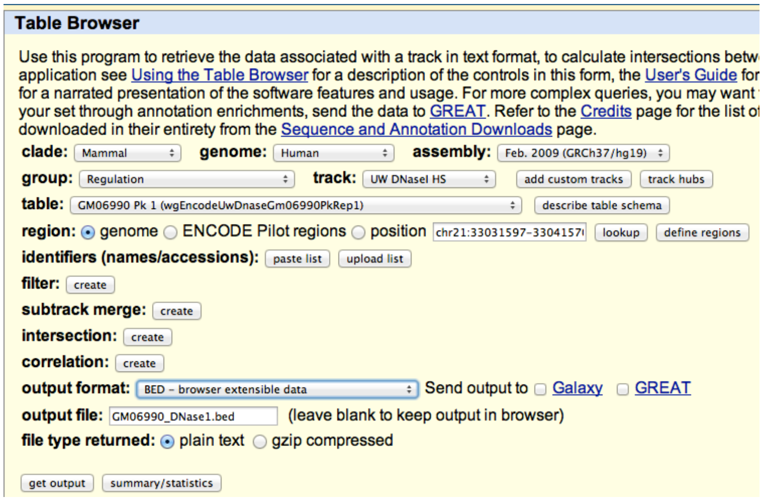

Figure 1: UCSC Genome Browser / Table Browser screen shot

500bp bin not overlap a feature in the BED file, then a background initiation probability is assigned to that bin, which is currently set to 10^{-4}.

To generate chromosome-specific IPLSs files from 'GM06990.bed', execute the command:

```
java -jar RepliconWrench.jar BedToIPLS -i GM06990.bed -o ipls -v 5
```

Where: '-i' specifies the input file; '-o' specifies the output directory and; '-v' specifies the column in the BED file on which to scale the probability of replication initiation (the fifth column in this case).

The end-result should be 22 directories (one per chromosome) under the 'ipls' directory. Each file should contain a chromosome-specific IPLS file.

4.1 BedToIPLS options

Here we provide a complete description of options available for the 'BedToIPLS' tool. It is possible to use this tool to generate IPLS files from most BED-formatted files.

Running

```
java -jar RepliconWrench.jar BedToIPLS
```

brings up the available options:

```
usage: BedToIPLS
 -b,--bin-width <arg>  specify bin width (default=500)
 -c,--chromosome <arg> chromosome column (1-based) default 1
 -e,--end <arg>        end column (1-based) default 3
```

```
-i,--input <arg>      input .bed file
-o,--out-dir <arg>    output directory
-s,--start <arg>      start column (1-based) default 2
-v,--value <arg>      value column (1-based) default 4
```

-i This required argument specifies the path to the BED file.

-b This required optional argument, if provided, specifies the genomic bin width for which to generate replication initiation probabilities. The default is 500bp bin (-b 500)– the setting used throughout the manuscript.

-c This optional argument specifies the column number in the input file containing the chromosome name. Default is '-c 1'.

-s This optional argument specifies the column number in the input file containing the genome feature start position. Default is '-s 2'.

-e This optional argument specifies the column number in the input file containing the genome feature end position. Default is '-e 3'.

-v This optional argument specifies the column number in the input file containing the value column, which will be scaled by 'RepliconWrench' to a probability of replication initiation. Default is '-v 4'.

5 Predicting and visualizing replication timing

The procedure outlined below illustrates how replication timing could be predicted for chromosome 14. Refer to 'runExample.sh' script for an illustration on how to extend this procedure to every chromosome using a simple for-loop.

Use the following command[3] to simulate replication timing for chromosome 14:

```
../replicon -nfork 95 -flowsorter 0.0,0.17,0.35,0.58,0.92,0.99,1.0
    ipls/chr14/chr14_GM06990 chr14_GM06990
```

The number of forks specified for this IPLS ('-nfork') is 95. This is calculated according to the regression formula presented in Figure 3D in the manuscript: $10.24 + 7.9^{-7} \times x$, where x is chromosome length (107,349,540 for chromosome 14). Once the simulation is complete, you should be able to see a file named 'chr14_GM06990.CGHnTiming.csv' that contains, for every 500bp genomic bin, measurements for how often that coordinate was placed in a particular flow

[3]You may see a message printed to screen informing you of a differing number of columns, please disregard it.

sorter gate bin according to DNA content (see the Methods section of the manuscript).

To generate a replication timing prediction file, execute:

```
perl genTimingFromCgh.pl chr14_GM06990.CGHnTiming.csv >
    chr14_GM06990.timing
```

The script 'genTimingFromCgh.pl' produces a file ('chr14_GM06990.timing') that assigns a replication time to each genome coordinate, where the first column is genome coordinate on chromosome 14 and second column is replication timing prediction.

Lastly, you could visualize (presented here in Figure 2) the replication timing profile for chromosome 14 of GM06990 cells, using R:

```
Rscript plotData.R chr14_GM06990.timing
```

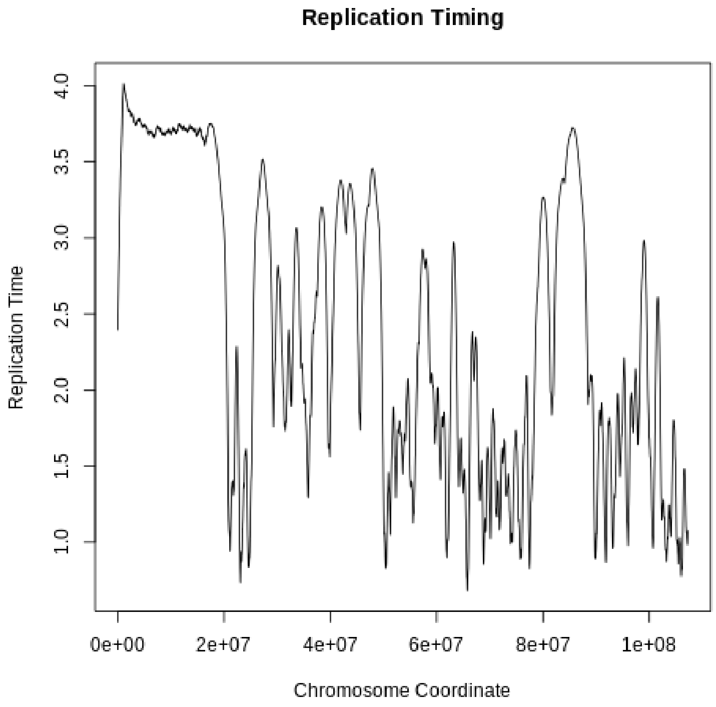

Figure 2: Replication timing prediction for chromosome 14 of GM06990 cells based on an IPLS generated from DNase I HS sites (y-axis; smaller values correspond to earlier time). Chromosome 14 coordinates are plotted on the x-axis

6 Replicon options

Here we provide details regarding options available when predicting replication timing with replicon.

Running Replicon without command-line options brings up a help message:

```
Usage: ../replicon [options] landscape out-prefix
Known options are
-flowsorter  Comma-separated list of flowsort boundaries
-ncells      Number of cells (default: 1000)
-nfork       Number of replication forks (default: 50)
-threads     Number of threads (default: 1)
```

These options are discussed in some detail below:

-flowsorter Biological experiments that measure replication timing rely on a flow-sorter to gate the cell population according to DNA content. Likewise, Replicon simulates a virtual flow-sorter. This option allows the user to manually specify the gate boundaries. If no boundaries are provided, then Replicon sets flow-sorter gates to be equidistant. In our experience (see manuscript) the following gate setting parameter produce optimal agreement between simulation and experiment:

```
-flowsorter 0.0,0.17,0.35,0.58,0.92,0.99,1.0
```

-ncells This setting specifies the number of virtual cells that Replicon will simulate. The default, if no argument is provided, is 1000 cells.

-nfork This setting specifies the number of replication forks that will be used to perform replication. We find that this setting is related to chromosome size according to the following relationship: $10.24 + 7.9^{-7} \times x$, where x is chromosome length (see Manuscript for details).

-threads This setting specifies the number of computer threads on which to run Replicon. Default is 1.
