# Supplementary figures and images for: A chromatin structure‐based model accurately predicts DNA replication timing in human cells
Source: Mol Syst Biol. 2014 Mar 28;10(3):722. doi: 10.1002/msb.134859 (PMC4017678; doi:10.1002/msb.134859)

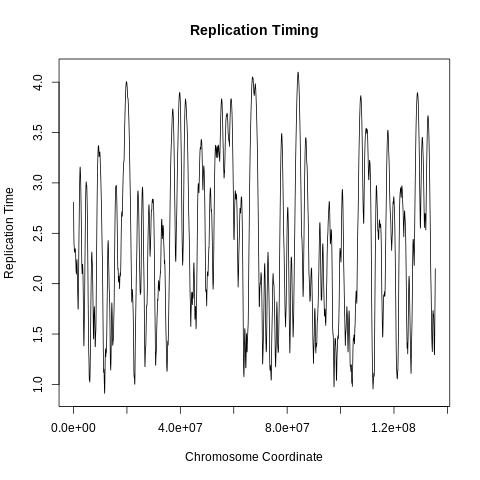

Supplement: Supplementary file 19 — Replicon software package [file MSB-10-3-722-s34.gz › replicon/example/replicationTiming/chr10_GM06990.CGHnTiming.csv.timing.gz_plot.png]

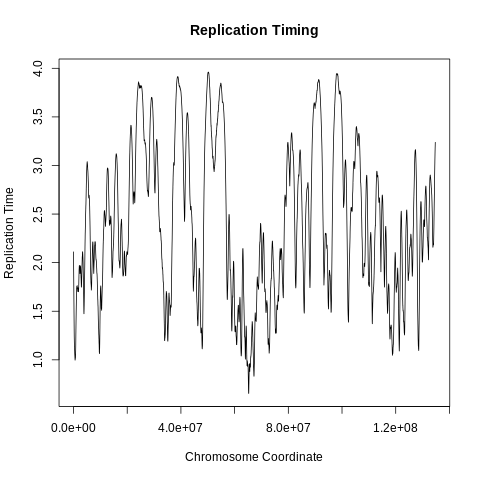

Supplement: Supplementary file 19 — Replicon software package [file MSB-10-3-722-s34.gz › replicon/example/replicationTiming/chr11_GM06990.CGHnTiming.csv.timing.gz_plot.png]

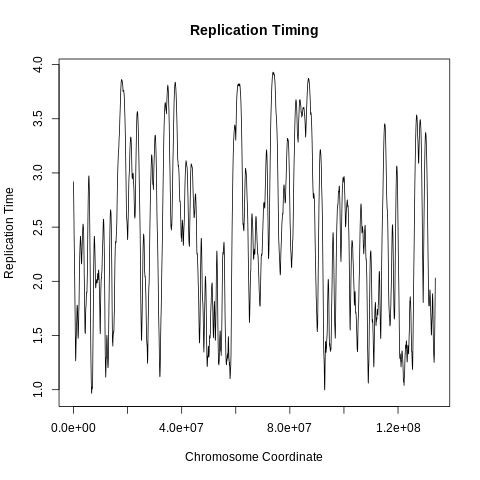

Supplement: Supplementary file 19 — Replicon software package [file MSB-10-3-722-s34.gz › replicon/example/replicationTiming/chr12_GM06990.CGHnTiming.csv.timing.gz_plot.png]

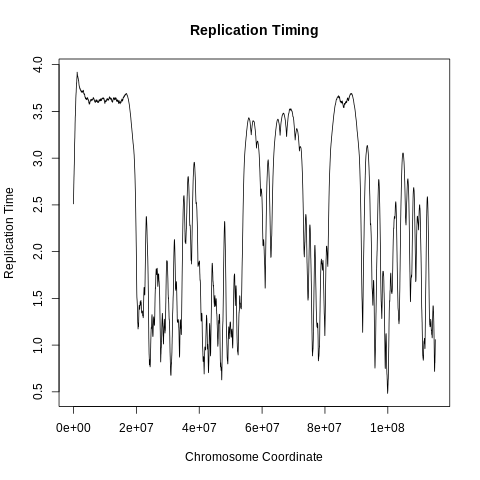

Supplement: Supplementary file 19 — Replicon software package [file MSB-10-3-722-s34.gz › replicon/example/replicationTiming/chr13_GM06990.CGHnTiming.csv.timing.gz_plot.png]

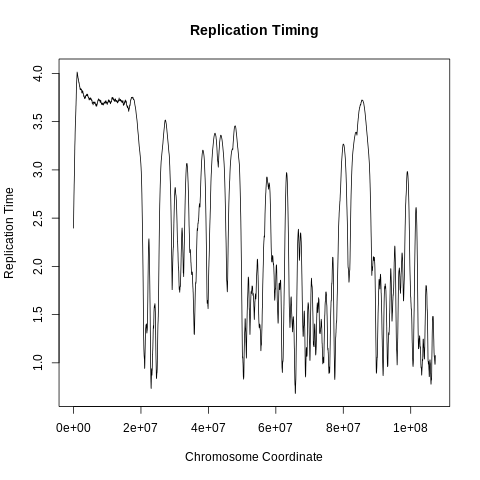

Supplement: Supplementary file 19 — Replicon software package [file MSB-10-3-722-s34.gz › replicon/example/replicationTiming/chr14_GM06990.CGHnTiming.csv.timing.gz_plot.png]

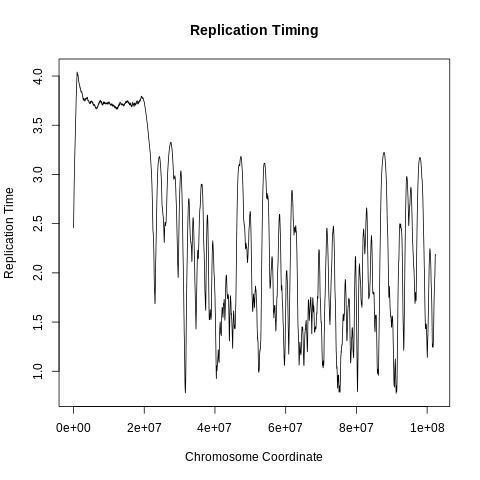

Supplement: Supplementary file 19 — Replicon software package [file MSB-10-3-722-s34.gz › replicon/example/replicationTiming/chr15_GM06990.CGHnTiming.csv.timing.gz_plot.png]

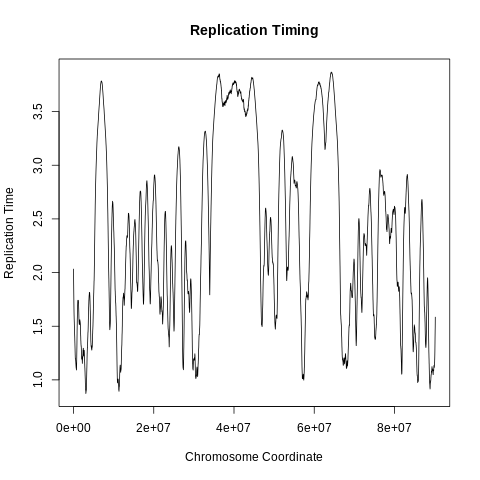

Supplement: Supplementary file 19 — Replicon software package [file MSB-10-3-722-s34.gz › replicon/example/replicationTiming/chr16_GM06990.CGHnTiming.csv.timing.gz_plot.png]

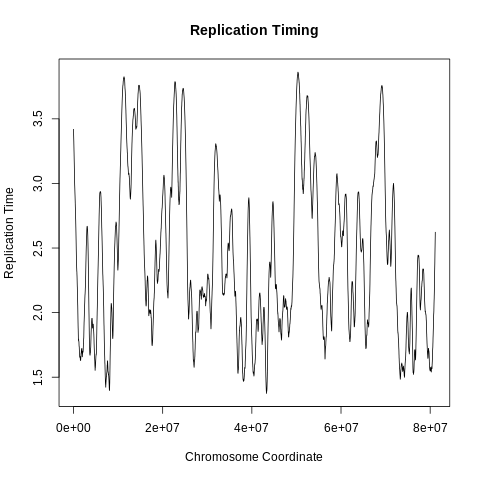

Supplement: Supplementary file 19 — Replicon software package [file MSB-10-3-722-s34.gz › replicon/example/replicationTiming/chr17_GM06990.CGHnTiming.csv.timing.gz_plot.png]

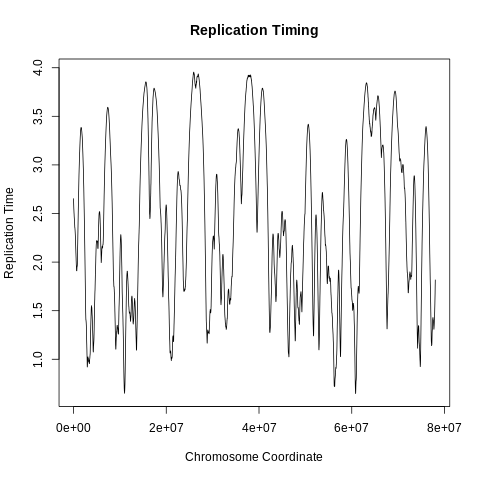

Supplement: Supplementary file 19 — Replicon software package [file MSB-10-3-722-s34.gz › replicon/example/replicationTiming/chr18_GM06990.CGHnTiming.csv.timing.gz_plot.png]

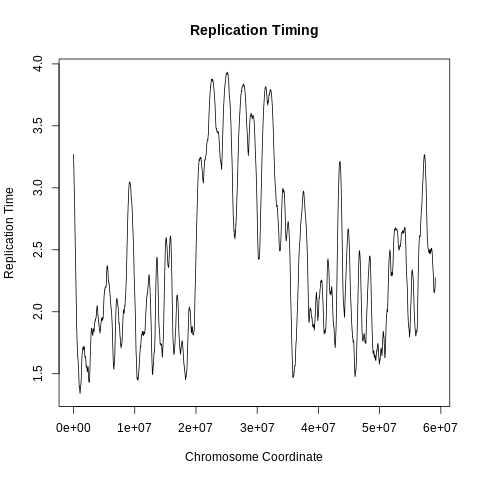

Supplement: Supplementary file 19 — Replicon software package [file MSB-10-3-722-s34.gz › replicon/example/replicationTiming/chr19_GM06990.CGHnTiming.csv.timing.gz_plot.png]

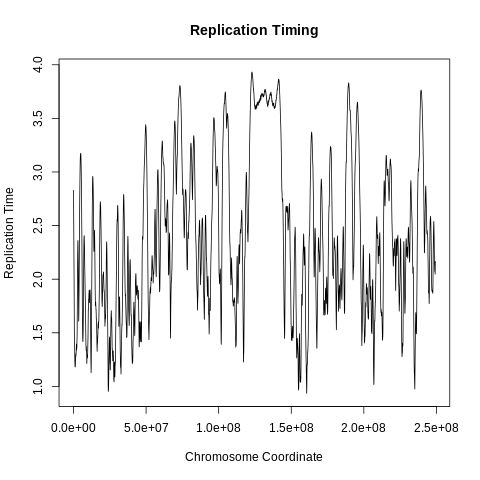

Supplement: Supplementary file 19 — Replicon software package [file MSB-10-3-722-s34.gz › replicon/example/replicationTiming/chr1_GM06990.CGHnTiming.csv.timing.gz_plot.png]

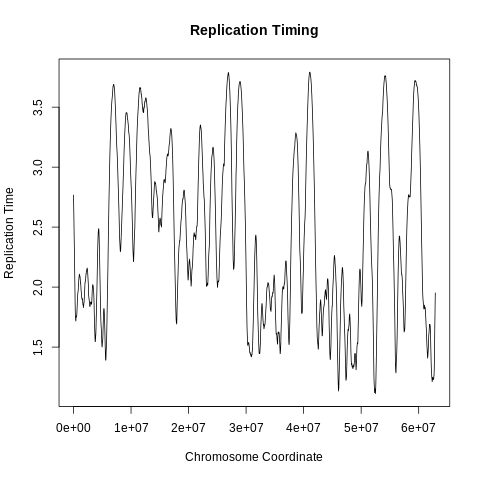

Supplement: Supplementary file 19 — Replicon software package [file MSB-10-3-722-s34.gz › replicon/example/replicationTiming/chr20_GM06990.CGHnTiming.csv.timing.gz_plot.png]

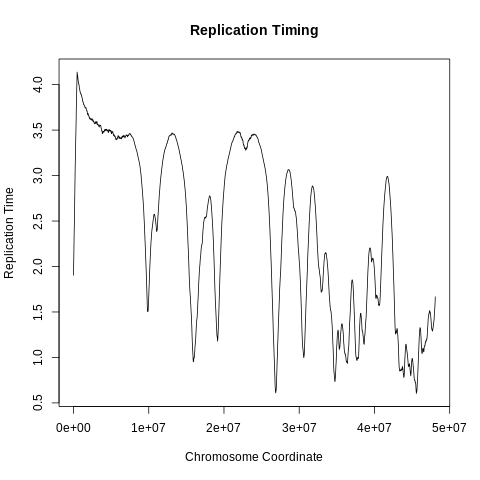

Supplement: Supplementary file 19 — Replicon software package [file MSB-10-3-722-s34.gz › replicon/example/replicationTiming/chr21_GM06990.CGHnTiming.csv.timing.gz_plot.png]

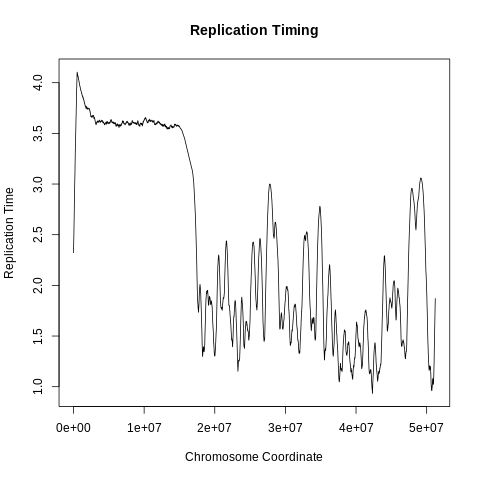

Supplement: Supplementary file 19 — Replicon software package [file MSB-10-3-722-s34.gz › replicon/example/replicationTiming/chr22_GM06990.CGHnTiming.csv.timing.gz_plot.png]

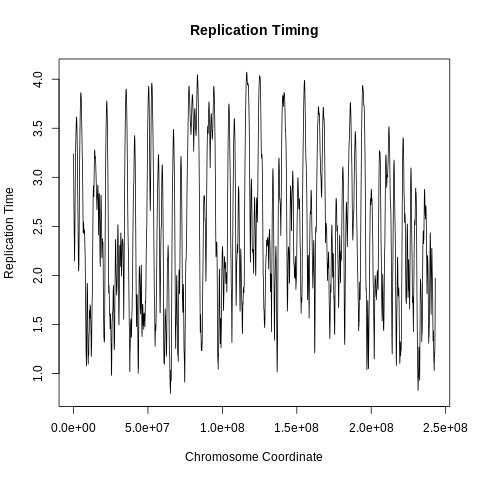

Supplement: Supplementary file 19 — Replicon software package [file MSB-10-3-722-s34.gz › replicon/example/replicationTiming/chr2_GM06990.CGHnTiming.csv.timing.gz_plot.png]

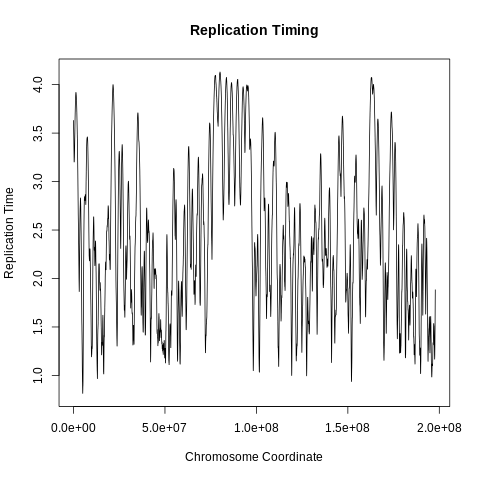

Supplement: Supplementary file 19 — Replicon software package [file MSB-10-3-722-s34.gz › replicon/example/replicationTiming/chr3_GM06990.CGHnTiming.csv.timing.gz_plot.png]

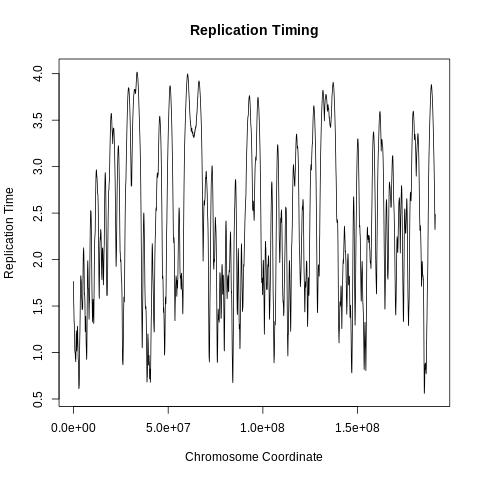

Supplement: Supplementary file 19 — Replicon software package [file MSB-10-3-722-s34.gz › replicon/example/replicationTiming/chr4_GM06990.CGHnTiming.csv.timing.gz_plot.png]

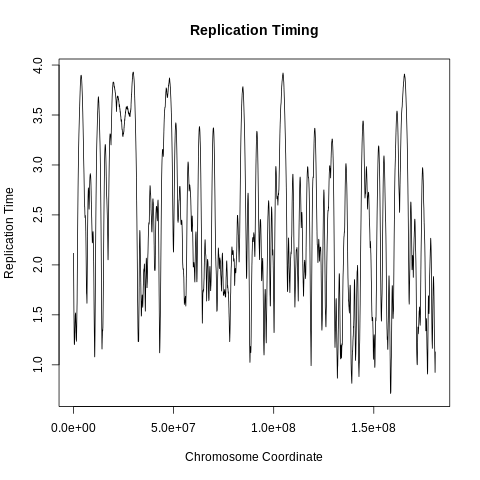

Supplement: Supplementary file 19 — Replicon software package [file MSB-10-3-722-s34.gz › replicon/example/replicationTiming/chr5_GM06990.CGHnTiming.csv.timing.gz_plot.png]

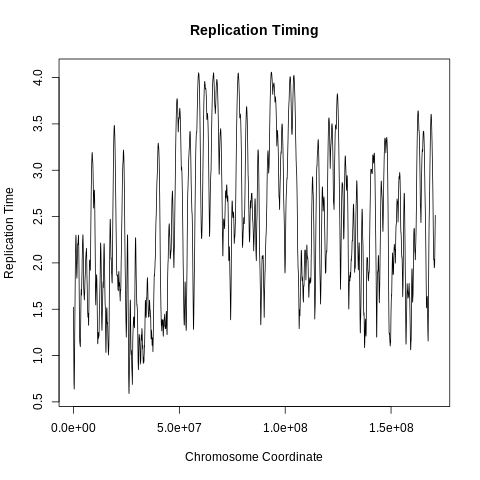

Supplement: Supplementary file 19 — Replicon software package [file MSB-10-3-722-s34.gz › replicon/example/replicationTiming/chr6_GM06990.CGHnTiming.csv.timing.gz_plot.png]

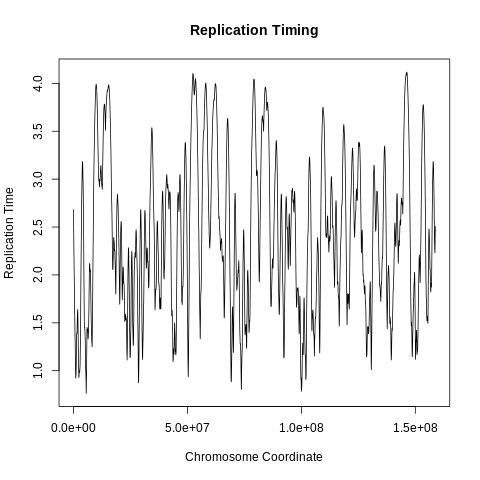

Supplement: Supplementary file 19 — Replicon software package [file MSB-10-3-722-s34.gz › replicon/example/replicationTiming/chr7_GM06990.CGHnTiming.csv.timing.gz_plot.png]

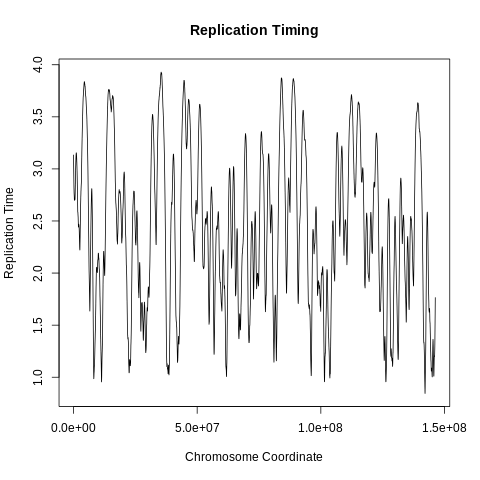

Supplement: Supplementary file 19 — Replicon software package [file MSB-10-3-722-s34.gz › replicon/example/replicationTiming/chr8_GM06990.CGHnTiming.csv.timing.gz_plot.png]

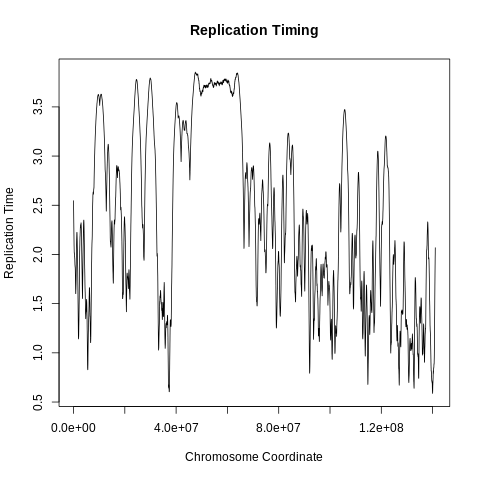

Supplement: Supplementary file 19 — Replicon software package [file MSB-10-3-722-s34.gz › replicon/example/replicationTiming/chr9_GM06990.CGHnTiming.csv.timing.gz_plot.png]
